# Supramolecular Assembly of Stepped‐Persistent ROS Nanogenerator for Sustained Tumor Immunotherapy

**DOI:** 10.1002/advs.75713

**Published:** 2026-05-15

**Authors:** Zhenqiang Wang, Ming Qin, Wenjing Lai, Shiwen Chen, Fengling Wang, Dandan Xie, Maohua Chen, Changpeng Hu, Kui Wang, Rong Zhang, Guobing Li

**Affiliations:** ^1^ Department of Pharmacy The Second Affiliated Hospital Third Military Medical University (Army Medical University) No. 83 Xinqiao Road Chongqing China

**Keywords:** DNA nanotechnology, Hybridization interface and assembly, Immunotherapeutic nanomaterials, Photodynamic therapy, Supramolecular polymers

## Abstract

ROS‐based tumor elimination could trigger systemic antitumor immune responses. However, these strategies are fundamentally restricted by insufficient ROS generation and uncontrollable kinetic processes. Besides, tumor‐associated macrophages often reverted to an immunosuppressive phenotype due to a lack of sustained stimulation. To achieve both effective tumor eradication and sustained activation of tumor immune microenvironment, a stepped‐persistent ROS nanogenerator supramolecularly assembled multiple ROS generation modules was synthesized. G‐quadruplexes (G4s)‐DNA nanochains, functionalized with Hemin and Ce6, served as a structure‐directing agent for synthesizing polydopamine nanofibers (PDANFs). Semiquinone radicals generated during dopamine polymerization were stabilized by supramolecular interaction between G4s and polydopamine oligomers. Under NIR irradiation, PDANFs could explosively produce singlet oxygen (^1^O_2_). Moreover, the nanoconfined catalytic cascade reaction between semiquinone radicals and G4s/hemin DNAzyme leads to a dissipative generation of hydroxyl radical (•OH), extending ROS generation after NIR irradiation above an effective cytotoxic level for adequate tumor inhibition and immune activation. Additionally, PDANFs also catalyzed pathological H_2_O_2_ to generate sub‐cytotoxic •OH, sustaining the antitumor phenotype of macrophage. The nanogenerators showed a slow reduction of ROS levels and remained above 47% and 23% after 1 and 48 h of photoactivation. Thereby, effective tumor inhibition and sustained immunoactivation were achieved. This system significantly advances ROS regulators for tumor immunotherapy.

## Graphical abstract

ROS catalytic systems often fail to achieve both effective tumor elimination and persistent immune activation due to their monotonous ROS production profiles. A stepped‐persistent ROS nanogenerator was developed by supramolecularly assembling multiple ROS generation modules within a nanofiber structure to simultaneously extend ROS generation duration above the effective cytotoxic threshold and sustain the stimulation of the antitumor phenotype of macrophages.

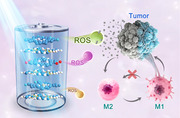

## 1 Introduction

Reactive oxygen species (ROS) play an essential role in physiological and pathological processes [1, 2]. In tumors, the impacts of ROS were highly complex and depended on ROS levels, intrinsic metabolic wiring, and cellular genetic components [3, 4, 5]. For instance, the increased ROS was associated with abnormal tumor cell growth, yet could exert cytotoxic effects when exceeding a threshold incompatible with cellular survival [6, 7, 8]. In addition, increased ROS in an appropriate range could directly activate the functions of immune cells within the tumor microenvironment (TME), while excessive ROS production led to immune cell dysfunction [9, 10, 11]. Although considerable achievements have been made in ROS‐based nanomedicines for tumor treatments, some fundamental but key questions, such as the rational design principle for balancing tumor inhibition and immune activation, have been held in low regard. Therefore, considering the different demands during tumor therapy, it was crucial to accurately control the ROS generation profiles.

From the perspective of tumor inhibition, a high concentration of ROS contributed to the oxidation damage of tumor cells and the subsequent release of damage‐associated molecular patterns (DAMPs) to activate innate immune responses [12, 13]. Photodynamic therapy (PDT) employing photosensitizers to absorb light energy and explosively generate ROS was often restricted by the limited irradiation duration and insufficient ROS generation in deep tumors [14, 15, 16]. ROS storage materials have gained increasing attention by incorporating energy storage modules and conversion modules that release ROS without light irradiation [17, 18]. Among them, the mutual synergy between energy storage and conversion modules for efficient ROS generation still remained a challenge. More importantly, multimodal combination strategies enabled the assembly of different approaches (e.g., photodynamic strategy and energy storage strategy), thereby further extending the duration of ROS generation above the effective cytotoxic threshold. However, the simple module superposition without spatiotemporal coordination led to a failure in synergistic enhancement of ROS production and a lack of controllable ROS generation profile [19, 20, 21]. Thus, the design of novel strategies with high energy conversion efficiency and a synergistic ROS generation profile of distinct modules was crucial for enhancing tumor eradication and immune activation.

From the perspective of immunomodulation of TME, ROS at sub‐cytotoxic levels could regulate immune cells [22, 23, 24, 25]. Tumor‐associated macrophages (TAMs) were the most abundant immune cells in the TME and were known to dampen antitumor immunity and promote tumor growth and metastasis [26, 27, 28]. Consequently, strategies using ROS that alter the TAMs phenotype away from its immunosuppressive state were expected to become the next generation of immunotherapies [29, 30]. However, current systems cannot simultaneously satisfy both high ROS stress for tumor eradication and low ROS stress for macrophage regulation. Moreover, the immunosupportive macrophages (M1) often reverted to immunosuppressive macrophages (M2) in the absence of sustained ROS stimulation [31]. Therefore, integrating different dynamics of ROS generation into a single system to achieve both tumor killing and long‐term maintenance of an immune‐supportive microenvironment was necessary.

**SCHEME 1:**
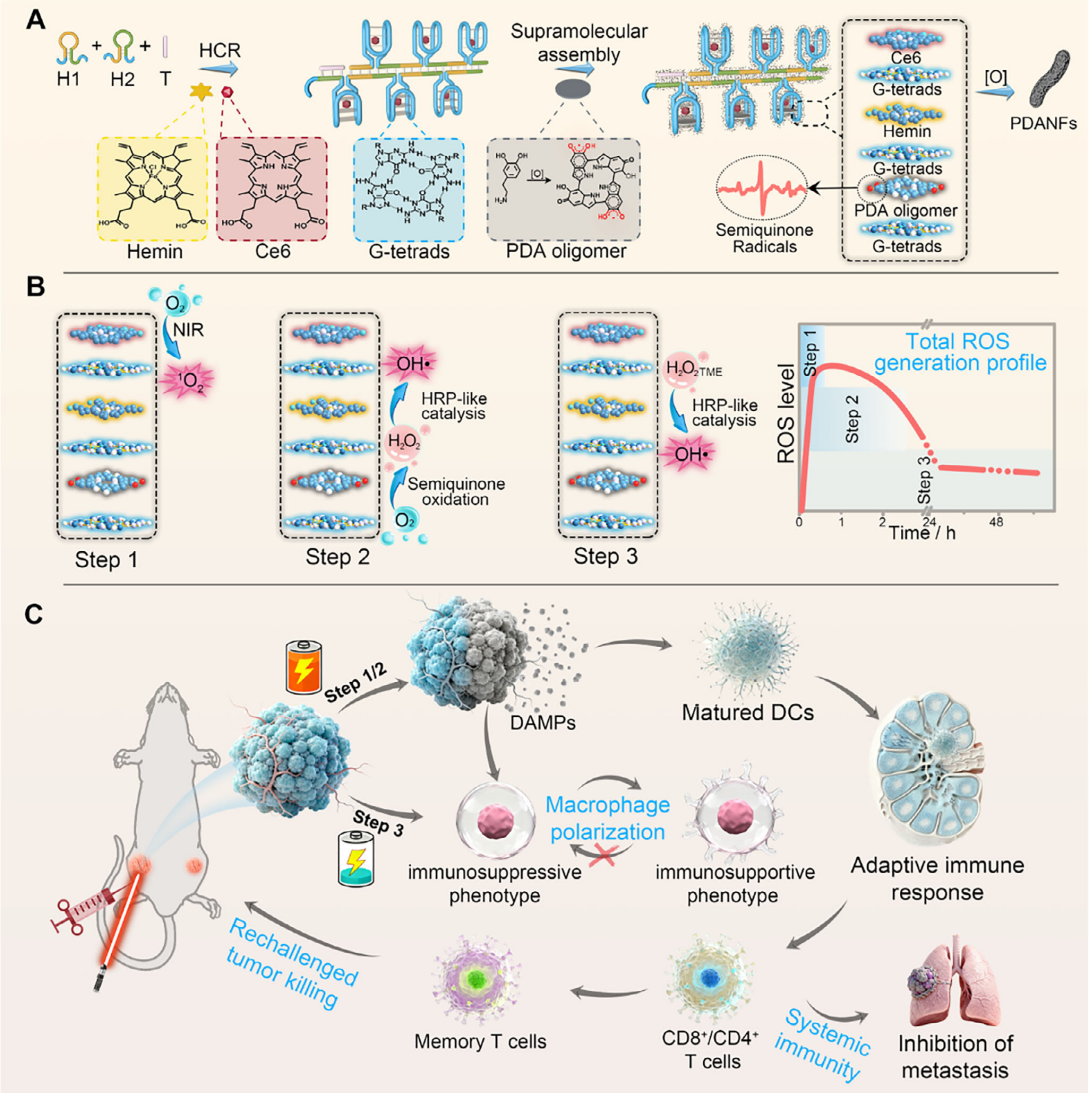
Schematic illustration of the design and application of PDANFs. (A) The synthesis mechanisms of the composite nanofiber‐based ROS nanogenerators. (B) The working principles of PDANFs for ROS generation. (C) The mechanism of PDANFs for improving tumor inhibition and maintenance of an immune‐supportive microenvironment.

Herein, a composite nanofiber with stepwise‐persistent ROS generation profiles was constructed for improving tumor immunotherapy. G‐quadruplexes (G4s)‐containing DNA nanochains assembled by a hybridization chain reaction (HCR) and loaded with hemin and Ce6‐ served as a structure‐directing agent for synthesizing polydopamine nanofibers (PDANFs). Semiquinone radicals were generated during the dopamine polymerization processes and stabilized by the supramolecular interactions between G4s and semiquinone radical‐containing polydopamine (PDA) oligomers through both steric protection and delocalization of spin density. Nanofibers could explosively produce a large amount of singlet oxygen (^1^O_2_) under NIR irradiation, and dissipatively generate hydroxyl radical (•OH) through a catalytic cascade reaction between semiquinone radicals and G4s/hemin DNAzyme. This nanoconfinement structure integrated the functions of distinct modules, thereby improving energy conversion efficiency, which in turn enhanced tumor eradication and potentiated immunogenic cell death (ICD)‐induced immune response. Besides, owing to their 1D morphology, PDANFs could effectively utilize the H_2_O_2_ in TME to continuously catalyze •OH generation, sustaining the antitumor phenotype of macrophages and achieving durable immunotherapy (Scheme 1).

## 2 Results and Discussion

### 2.1 Characterization of Supramolecular Assembly and Interface‐Oriented Synthesis

The G4s‐containing DNA nanochains were first prepared by an HCR amplification response with a toehold strand (T) and two amplification hairpins (H1 and H2). Specifically, T hybridized with domain I (green color) of H1 through the toehold‐mediated strand displacement (TMSD), thereby exposing domain II (yellow color) of H1 (step 1) (Figure S1). The exposed domain II was then hybridized with domain I’ (yellow color) of H2, completely exposing domain II’ (green color) in H2 (step 2). The exposed domain II’ subsequently hybridized with domain I (green color) (step 3), activating autonomous cross‐opening of H1 and H2 through the TMSD cascade (steps 2 and 3). This process leads to the self‐assembly of DNA constructs composed of alternating G4s on both sides of the nanochains. In the presence of Hemin and Ce6, the resulting DNA nanochains were anticipated to exhibit horse‐radish peroxidase (HRP)‐mimicking H_2_O_2_‐mediated oxidation ability and photodynamic activity.

**FIGURE 1:**
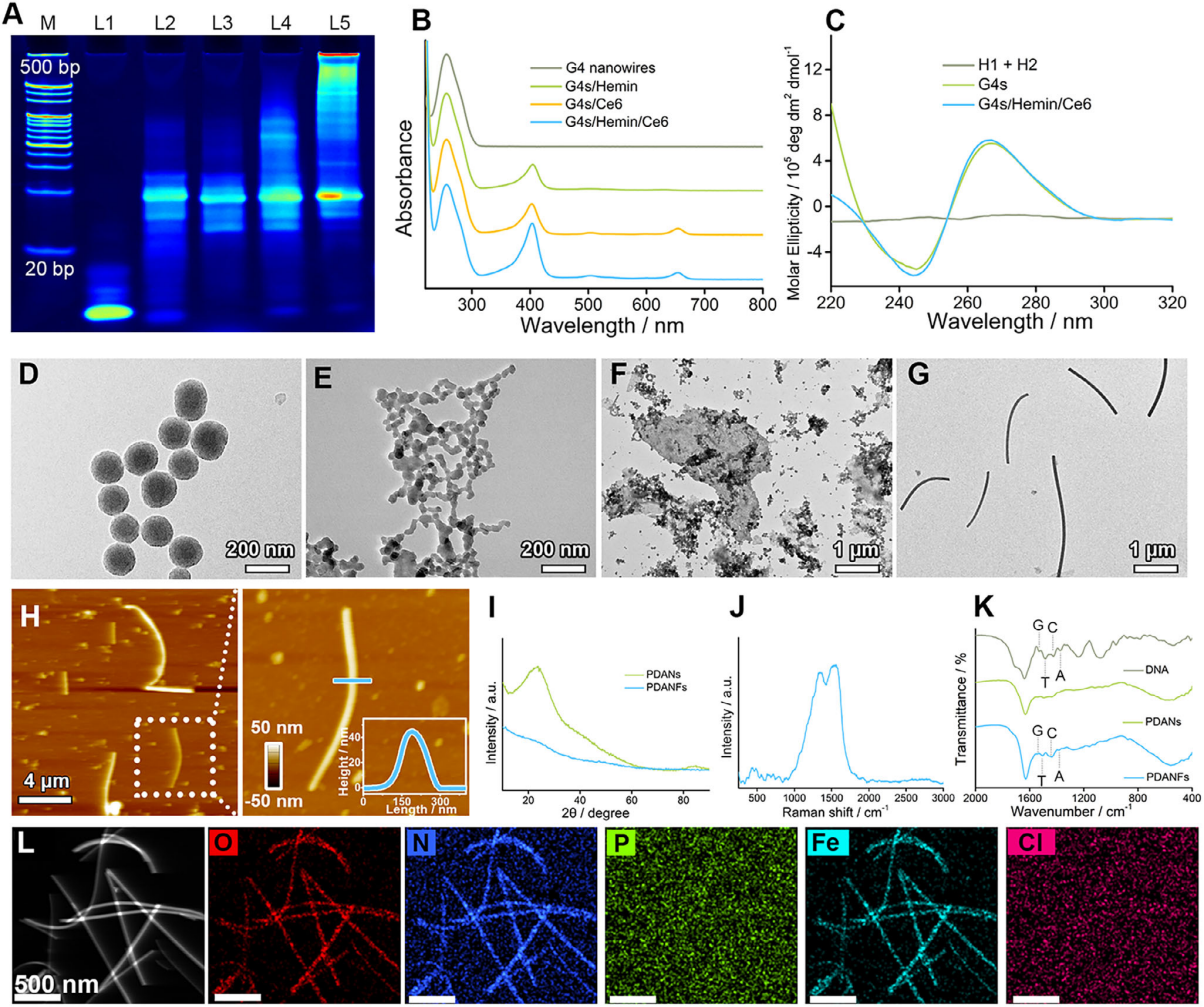
Characterization of PDANFs. (A) Electrophoretic characterization of the toehold‐guided HCR‐generated G4 nanochains. Lane M: DNA marker; Lane1: T DNA; Lane 2: H1; Lane 3: H2; Lane 4: H1/H2 (1:1); Lane 5: T/H1/H2 (1:200:200). (B) UV–vis spectra of G4 nanochains, G4s/Hemin complexes, G4s/Ce6 complexes, and G4s/hemin/Ce6 complexes. (C) CD spectra of H1/H2, G4 nanochains, and G4s/hemin/Ce6 complexes. TEM images of PDANs (D), CH‐PDANs (E), DNA‐PDANs (F), and PDANFs (G). (H) AFM images and height distribution based on the blue line in the selected nanosheets. (I) XRD spectra of PDANs and PDANFs. (J) Raman spectra of PDANs and PDANFs. (K) FT‐IR spectra of DNA, PDANs, and PDANFs. (L) HRTEM images showing the morphology and composition of PDANFs, including the bright‐filed TEM images, a dark‐filed TEM image, and the corresponding element mapping data.

The formation of the HCR‐mediated G4s nanochains was first investigated by native‐PAGE. Gel electrophoresis found that T, H1, and H2 DNA showed a signal band in lanes 1–3, respectively. After mixing H1 with H2 in the annealing buffer, additional bands with lower mobility were shown in lane 4, which might be caused by the nonspecific assembly between H1 and H2. More importantly, after HCR amplification (lane 5), most DNA products were trapped in the gel well, confirming that the T strand could efficiently initiate HCR of H1 and H2 for generating DNA nanochains (Figure 1A). Besides, both the typical absorption peaks of Hemin and Ce6 were observed in the UV–vis spectrum, indicating successful conjugation of Hemin and Ce6 to the G4s nanochains (Figure 1B). Moreover, circular dichroism (CD) spectrometry was used to verify the formation of G‐quadruplex structures in the HCR products. Compared with the unassembled hairpin strands, G4‐containing nanochains and the G4s/Hemin/Ce6 complexes showed prominent peaks at 265 nm (Figure 1C). Together, these results indicated that the HCR products could form parallel G4 structures, capable of binding Hemin and Ce6.

Next, G4s/hemin/Ce6 complexes as structure‐directing agents dispersed in aqueous solution were mixed with dopamine together with tris(hydroxymethyl)‐aminomethane (Tris) as a catalyst. Transmission electron microscopy (TEM) images of synthetic products prepared by different structure‐directing agents are shown in Figure 1D–G. First, polydopamine nanoparticles (PDANs) prepared without a structure‐directing agent showed a spherical morphology with a diameter of 100–150 nm. Second, CH‐PDANs prepared by adding Hemin/Ce6 complexes in dopamine solution exhibited smaller spherical particles with an average diameter of 30 nm. Moreover, DNA‐PDANs prepared using linear DNA (salmon sperm DNA, ∼2000 bp) lacking hairpin and G4 structures, were dominantly composed of tiny nanoparticles with sizes of 50 nm and nanosheets with a micro‐scale lateral size (up to 5 µm). The formation of nanosheets was attributed to the vertical interdigitation and lateral packing of PDA's primary planar structures on DNA structures [32]. However, a quite different morphology of nanofibers was obtained when G4s/hemin/Ce6 complexes were used during synthesis. The resulting nanofibers exhibited longitudinal lengths up to 10 µm and diameters of 40–80 nm (Figure 1G,H). Clean nanofibers were observed at the dopamine/G4 nanochains weight ratio of 1:0.125 (Figure S2). Both the higher‐ and lower‐concentrations of G4s/hemin/Ce6 complexes led to the generation of small nanoparticles/sheets. Therefore, the dopamine/G4 nanochains weight ratio of 1:0.125 was used for further research.

Powder X‐ray diffraction (XRD) pattern of PDANs revealed a broad peak at a 2θ value of 24.6°, which was related to the intrinsic layer distance of 0.36 nm from the *π–π* stacking spacing of PDA’ primary structure of planar tetramers [32]. However, the diffraction peak disappeared in PDANFs’ pattern, likely due to PDA oligomers bonding to both the backbone (DNA duplex) and the side chains (G‐quadruplexes) of G4 nanowires. This anisotropic binding led to the disappearance of characteristic diffraction peaks in the XRD (Figure 1I). Raman spectroscopy further confirmed successful polymerization of PDANFs (Figure 1J). Moreover, Fourier transform infrared (FTIR) spectroscopy (Figure 1K) and high‐angle annular dark‐field scanning TEM‐energy‐dispersive X‐ray spectroscopy (HAADF‐STEM‐EDS) elemental mapping (Figure 1L) were applied to identify the existence of Hemin and DNA molecules. Characteristic FTIR peaks of cytosine, adenine, thymine, and guanine nitrogenous bases appeared at 1373, 1423, 1484, and 1529 cm^−1^, respectively. HAADF‐STEM‐EDS elemental mapping also revealed a distribution of Fe and P elements, confirming successful incorporation of Hemin and DNA into the PDANFs.

**FIGURE 2:**
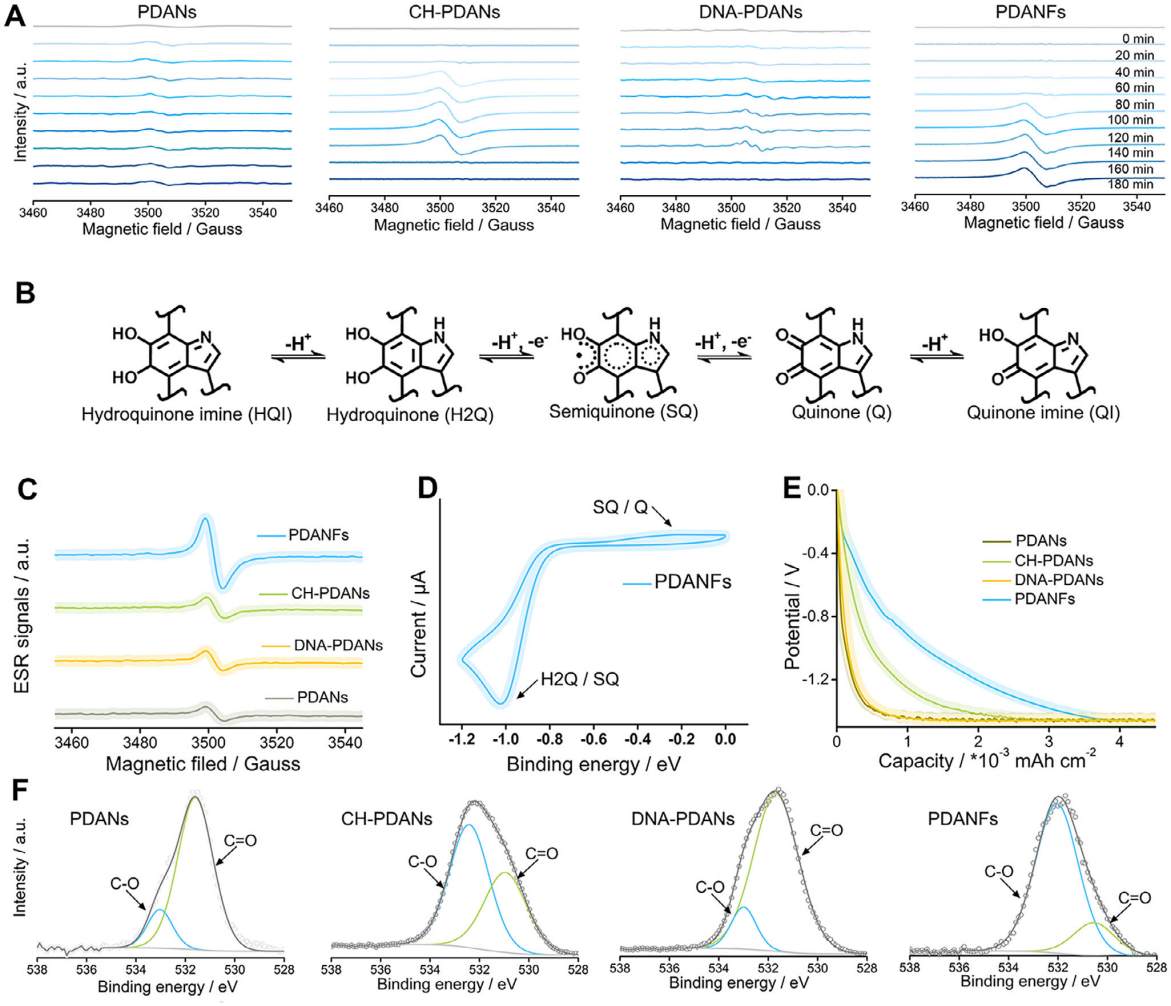
Host–guest supramolecular interaction studies between G4 nanochains with semiquinone radicals and proposed formation mechanism of PDANFs. (A) Evolution of the ESR spectra of the synthesis solutions. (B) Initial steps of dopamine autoxidation. (C) ESR spectra of different products. (D) CV of PDANFs film‐coated glassy carbon electrode (GCE) in 0.1 m Bu_4_NPF_6_ acetonitrile solution at 0.1 V s^−1^. (E) Galvanostatic discharge curve of different sample‐coated electrodes with a current of different sample‐coated electrodes with a current of 0.1 µA in 0.5 m Na_2_SO_4_ buffer (pH 7.0). (F) High‐resolution XPS spectra of O1s of PDANs, CH‐PDANs, DNA‐PDANs, and PDANFs.

To further elucidate the formation mechanisms, a series of characteristic experiments was conducted. First, the time‐dependent electron spin resonance (ESR) spectra of the reaction solutions were recorded (Figure 2A). The dopamine solution without the addition of a structure‐directing agent showed a weak peak of the PDA semiquinone radicals within 140 min, which gradually decreased over time. Semiquinone radicals were formed, contributing to a redox reaction, which has been proposed as the main source of mobile electronic charge carriers in eumelanin (Figure 2B) [33, 34]. However, semiquinone radicals were gradually consumed by further oxidation because they were highly reactive species undergoing single‐electron redox processes. Upon the addition of Ce6/Hemin complexes and DNA, the peak intensity of semiquinone radicals increased to varying extents, which was probably due to *π–π* stacking interaction between the semiquinone‐containing PDA oligomers with Ce6/Hemin complexes and DNA bases. Nonetheless, the semiquinone signals also decreased due to the lack of effective protection. After the addition of G4s/hemin/Ce6 complexes, the ESR signals of the dopamine solution were markedly enhanced and sustained within 180 min. The self‐polymerization products were collected after 24 h of reaction and stored in N_2_‐saturated water, respectively. PDANFs suspension exhibited a significant semiquinone peak, which suggested that the stable semiquinone radicals were formed during the polymerization process (Figure 2C).

To verify the protective effect of semiquinone radicals by G4 nanochains, cyclic voltammetry (CV) experiments were performed. As shown in Figure 2D and Figure S3, PDANs, CH‐PDANs, and DNA‐PDANs showed a reversible one‐electron reduction process typical of quinone (*E*_1/2_ = −0.86 V). When the CV experiment was performed on PDANFs, the corresponding oxidation peak at −0.76 V disappeared and was replaced by a new oxidation peak at −0.25 V. Hemin, Ce6, and DNA have no redox properties in the electrochemical window between −1.2 and 0 V. The large anodic shift (510 mV) provided direct evidence for the thermodynamic stabilization of the complexed semiquinone radicals. Moreover, the electron storage capacity of different nanomaterials was measured through galvanostatic discharge measurements. The results showed that the PDANFs‐coated electrode stored more electrons for discharging than PDANs, CH‐PDANs, and DNA‐PDANs, suggesting that the PDANFs contained a higher proportion of semiquinone or catechol moieties capable of storing electrons in a donatable state (Figure 2E).

Furthermore, X‐ray photoelectron spectroscopy (XPS) analysis was performed, and detailed chemical composition was acquired from the deconvoluted spectra of O1s regions. The O1s region was deconvoluted into two components assigned to O═C (∼531.0 eV) and O─C (∼532.9 eV) species. Higher content of O─C (83) in PDANFs was observed, as compared to that of PDANs (15%), CH‐PDANs (59%), and DNA‐PDANs (23%). The elevated O─C fraction was attributed to the retention of the hydroquinone and semiquinone species during the dopamine polymerization, further supporting the interaction between PDA oligomers and G4 nanochains (Figure 2F).

By decoding the chemical structure of G4 nanochains and PDA oligomers, and analyzing the characterization results above, some important features that direct strong supramolecular interactions were found. The sandwich structure of G4s was generally composed of stacked arrays of G‐tetrads, each self‐assembled by purine rings through hydrogen bonding. The purine ring is a planar N heterocycle, which can act as a π acceptor to interact with a strong π donor, such as a PDA oligomer. Semiquinone radicals‐containing oligomers generated via the dismutation of the intermediates of dopamine oxidation were “captured” in the interplane spaces of G‐quadruplexes and stabilized by the steric protection of G4s, which provided powerful evidence for the supramolecular interaction between G4s and PDA oligomer (Figure S4). Therefore, G4 nanochains containing repetitive G4s units could act as deposition sites for PDA synthesis, thereby enabling the synthesis of semiquinone radical‐containing PDA nanofibers.

To validate the synthesis model, molecular simulation experiments were conducted (Figure S5). First, molecular docking simulation results indicated that the PDA oligomer and G4s could form a high‐affinity conformation. To evaluate the overall binding stability, the time‐dependent root‐mean‐square deviation (RMSD) during molecular dynamics simulations was analyzed. The results indicated that after a brief period of conformational adjustment (0–5 ns), the system gradually entered a relatively stable state, with the RMSD notably decreasing and stabilizing within the range of 2.3–3.0 Å. Over the 100 ns simulation, the overall RMSD of the complex remained within a stable range without persistent drift or divergence. Moreover, the solvent‐accessible surface area (SASA) results were consistent with the RMSD data, with the SASA remaining within the range of 4050–4200 Å^2^, exhibiting relative stability alongside some thermal fluctuations. Furthermore, binding free energy decomposition was performed using the MM‐PBSA method. The gas‐phase energy (ΔG gas) was −110.22 kcal/mol, indicating highly favorable interactions between the PDA oligomer and G4s and demonstrating a strong binding driving force. Further decomposition revealed that both van der Waals interactions (VDWAALS, −59.64 kcal/mol) and electrostatic interactions (EEL, −50.57 kcal/mol) were negative and contributed comparably, indicating that the complex was driven by both hydrophobic and electrostatic interactions. The total binding free energy (ΔG total) was −31.90 kcal/mol, indicating a high affinity between the ligand and the receptor.

### 2.2 Catalytic Studies of Stepped‐Persistent ROS Generation

**FIGURE 3:**
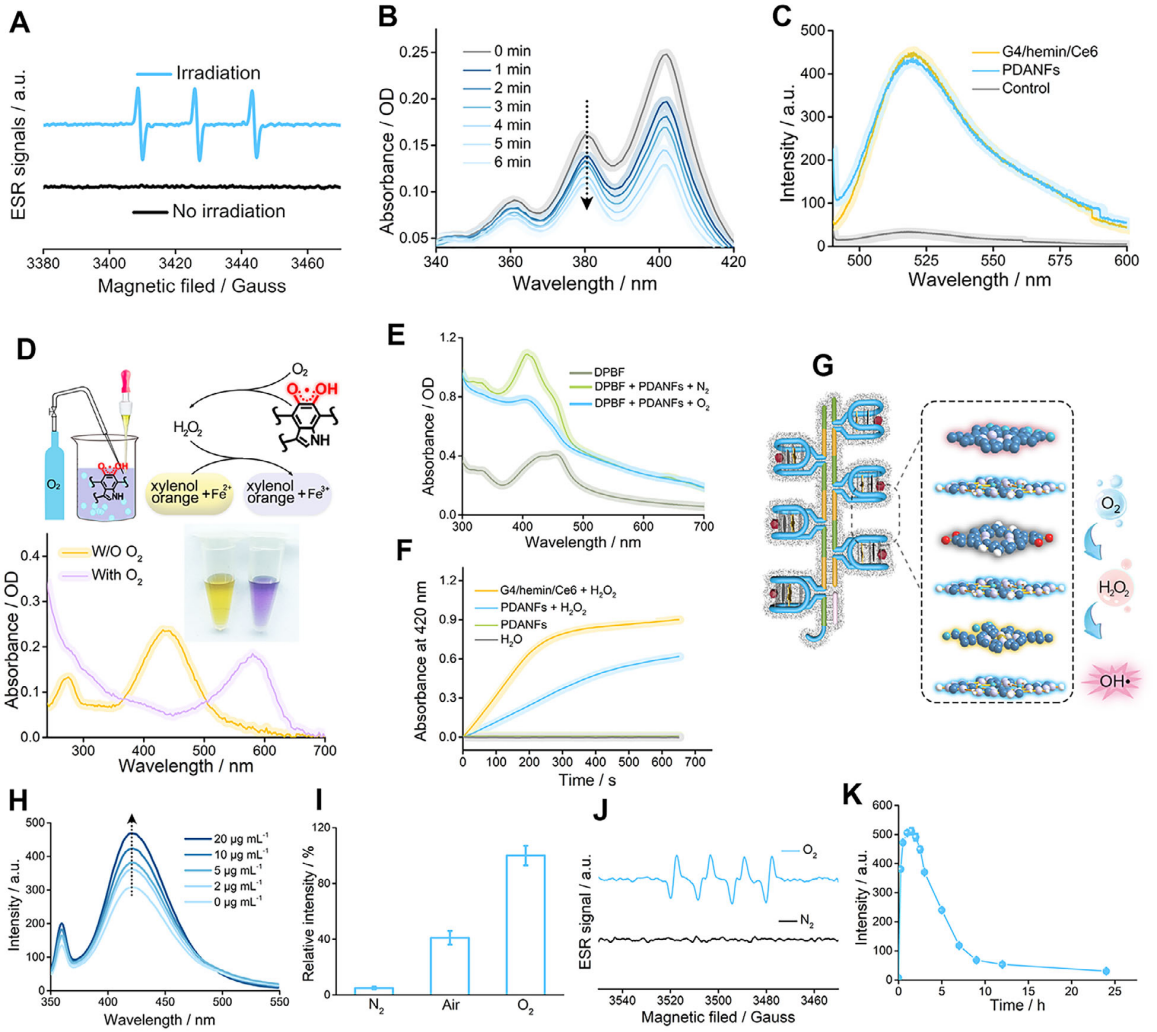
Catalytic cascade studies of PDANFs for stepped‐persistent ROS generation. (A) ESR spectra of PDANFs with or without NIR irradiation (660 nm, 3 min) using TEMP as the free radical‐scavenging agent. (B) UV–vis absorbance spectra of ABDA solutions (10 µM) irradiated under NIR irradiation (660 nm) in the presence of PDANFs (10 µg mL^−1^). (C) UV–vis absorbance spectra of SOSG solution (5 µM) upon NIR irradiation. G4s/hemin/Ce6 complexes (4 µg mL^−1^) and PDANFs (20 µg mL^−1^) with the same iron stoichiometry according to the ICP measurements were used in this experiment. (D) Schematic diagram of H_2_O_2_ detection by the H_2_O_2_ assay kit. Digital image and UV–vis spectra of detection solutions. (E) UV–vis spectra of DPBF solution (100 µM) upon addition of PDANFs (10 µg mL^−1^) and bubbling different gases. (F) Time‐dependent absorbance variations of ABTS (1.6 mM) at 420 nm in the HRP‐like catalysis system upon addition of H_2_O_2_ (1 mM). (G) Proposed mechanism of the cascade reaction of PDANFs for dissipative generation of •OH. (H) Fluorescence spectra of TA solution (0.5 mM) in the presence of different concentrations of PDANFs. (I) Relative fluorescence intensity of TA solution in the presence of PDANFs under different atmospheres. (J) DMPO spin‐trapping ESR spectra recorded for •OH in PDANFs suspension under different atmosphere. (K) •OH generation profile of PDANFs (10 µg mL^−1^) detected by DCFH (n = 3).

The stepwise and sustained ROS generation capability of PDANFs was systematically investigated. First, the photodynamic efficacy of PDANFs was determined by using different methods. As shown in ESR spectra, the characteristic 1:1:1 triple signal of the 2,2,6,6‐tetramethyl‐1‐piperidinyloxyl (TEMPO) free radical, derived from the spin‐trapping agent (TEMP, 2,2,6,6‐Tetramethypiperidine) for ^1^O_2_, appeared in the presence of PDANFs plus NIR irradiation (Figure 3A). Moreover, the absorbance of 9,10‐anthracenediylbis(methylene)dimalonic acid (ABDA) gradually decreased in a time‐dependent manner after treatment with PDANFs plus NIR irradiation (Figure 3B). Consistently, after treatment with PDANFs plus NIR irradiation, the fluorescence intensity of SOSG was significantly enhanced, reaching a comparable level to that observed with G4s/Ce6 (Figure 3C). These results collectively illustrated that the photodynamic activity of Ce6 was well preserved during the synthesis process.

Given the redox reactivities, semiquinone radicals could donate electrons to the oxidant O_2_ to generate H_2_O_2_ or O_2_˙ˉ. To verify this process, a H_2_O_2_ assay kit was employed, which was based on the oxidation of ferrous iron (Fe^2+^) to ferric iron (Fe^3+^) by peroxides, and the Fe^3+^ then combines with a dye‐xylenol orange to form a purple‐colored complex with the maximum at 580 nm measurable. As shown in Figure 3D, incubation of PDANFs with the detection solution under sufficient O_2_ supply resulted in an obvious color change from yellow to purple and a red shift in absorption maximum, confirming H_2_O_2_ generation. Besides, for O_2_˙ˉ detection, 1,3‐diphenylisobenzofuran (DPBF) was utilized as a chemical probe because it could be oxidatively degraded in the presence of O_2_˙ˉ with a gradual decline of its absorption at 410 nm. After adding PDANFs, the DPBF solution displayed a remarkably higher absorption, which was contributed by the broad‐spectrum absorption of polydopamine (Figure 3E). However, an obvious decrease in absorption intensity was observed upon bubbling O_2_, indicating that O_2_˙ˉ was generated in this process. The unstable O_2_˙ˉ was then readily dismutated to H_2_O_2_, which subsequently acts as a substrate for the next stage of HRP reaction (Figure S6).

Next, the standard peroxidase‐mimicking oxidation of 2,2’‐azino‐bis(3‐ethybenzthiazoline‐6‐sulfonic acid) (ABTS) was used for monitoring the HRP‐like oxidizing activity of PDANFs toward exogenously added H_2_O_2_, since the reaction produced a green oxidized product ABTS˙^+^ with a characteristic absorbance peak at 420 nm. To eliminate interference from semiquinone‐mediated H_2_O_2_ formation, the experiments were conducted using O_2_‐treated PDANFs (PDANFs‐ox) to pre‐consume the semiquinone radicals. Presumably, the catalytic sites of G4s/hemin/Ce6 complexes were covered by the PDA, which could result in reduced catalytic activity toward exogenously added H_2_O_2_. However, PDANFs exhibited a slightly reduced catalytic activity than G4s/hemin/Ce6 complexes (Figure 3F), which might be caused by the 1D morphology promoted exposing of catalytic sites.

To further determine the effect of morphology on catalytic activity, a G‐quadruplexes‐directed spherical nanoparticles (PDANSs) was synthesized. In detail, single‐stranded nucleic acid structures containing repetitive G‐quadruplex units were synthesized through a rolling circle amplification (RCA) reaction. In contrast to the rigid rod‐like structure of double‐stranded nucleic acids, single‐stranded nucleic acids exhibit a flexible, coiled conformation. As a result, when used as structure‐directing agents, they promote the formation of spherical nanoparticles (Figure S7A). We compared the catalytic activity of PDANFs‐ox and PDANSs‐ox toward exogenously added H_2_O_2_. The results revealed that, at equivalent mass, PDANSs‐ox exhibited markedly lower catalytic activity, approximately 35% of that observed for PDANFs‐ox (Figure S7B). This reduction was likely due to G4/hemin complexes being encapsulated inside the nanospheres, limiting their accessibility to the substrate.

Then, HRP‐like oxidizing activity of PDANFs toward endogenously generated H_2_O_2_ (produced via semiquinone‐oxidation) was also detected (Figure 3G). The cascade process was verified by using terephthalic acid (TA) as signal probe, which could capture •OH and generate fluorescent 3‐hydroxy terephthalic acid (TAOH). As shown in Figure 3H, the fluorescence intensity of the O_2_‐staturated TA solution at 425 nm was increased with higher PDANFs concentrations, confirming the generation of •OH. Moreover, the fluorescence intensity of TA solutions incubated with PDANFs was recorded under different atmospheres. As shown in Figure 3I, bubbling N_2_ into the mixture did not cause a noticeable fluorescence increase, while O_2_ induced a drastic enhancement of fluorescence intensity. Compared to the O_2_‐treated group, 40% fluorescence increase was observed in normoxic conditions due to the relatively lower O_2_ content (∼21%), which proved the importance of O_2_ as a substrate participating in the cascade reaction. ESR spectroscopy using 5,5‐dimethyl‐1‐pyrroline N‐oxide (DMPO) as trapping agent further validated this process, revealing the characteristic DMPO‐HO• signal in the presence of PDANFs and O_2_ (Figure 3J). These results collectively confirmed the •OH‐involved cascade reaction mediated by PDANFs.

Given that ROS generation in both the photodynamic stage and the catalytic cascade stage requires O_2_, and that O_2_ levels are relatively low in tumor tissues, the effect of varying O_2_ concentrations on ROS production by PDANFs was further investigated. As reported, breast cancer is characterized by pathological hypoxia, with an average oxygen partial pressure of 10.0 mmHg in tumor tissues [35]. To simulate this condition, PDANFs dispersion (pH 6.5) was treated with N_2_ containing 0.1%, 1%, 2%, 5%, 10%, and 21% O_2_, achieving oxygen partial pressures of 0.71, 7.1, 14, 36, 71, and 150 mmHg, respectively. Among these, dispersion solution treated with 1%–2% O_2_ was used to mimic the hypoxic tumor microenvironment, while those exposed to other oxygen concentrations served as controls. The results showed that at 1% O_2_ concentration, the production efficiencies of ^1^O_2_, H_2_O_2_, and •OH by PDANFs were 39% (Figure S8A), 64% (Figure S8B), and 41% (Figure S8C), respectively, of those observed at 21% O_2_ concentration. Moreover, we assessed the •OH generation during the catalytic cascade reaction using 2’,7’dichlorodihydrofluorescein (DCFH) as a probe. As shown in Figure 3K, upon exposure to O_2_ (1%), ROS generation by PDANFs peaked at 1.5 h and then declined due to the limited electron storage capacity of semiquinone radicals, retaining 47% and 23% of peak activity at 5 and 7 h, respectively.

To evaluate the stability and lifetime of the radicals in the PDANFs dispersed in N_2_‐saturated aqueous media, •OH generation efficiency was monitored over time. As shown in Figure S9, the catalytic activity of PDANFs showed negligible reduction (only 20% decline) at 20 days and remained above 50% after 60 days of storage in N_2_‐saturated aqueous media. Moreover, the increased O═C species (from 17% to 38%) confirmed the oxidation of semiquinone radicals for the O_2_‐treated PDANFs (Figure S10). Furthermore, PDANFs synthesized at a dopamine/G4 nanowires mass ratio of 1:0.125 exhibited the highest ROS‐producing capacity across all stages (Figure S11). Additionally, the structural stability of the nanofibers was evaluated by dispersing them in fetal bovine serum (FBS) for 30 days and assessing changes in size and morphology. As shown in Figure S12, no significant alterations in size or morphology were observed, indicating that PDANFs possess favorable structural stability under physiological conditions.

In summary, the results above indicated that PDANFs exhibited photodynamic activity for explosive generation of ^1^O_2_, cascade catalytic activity for dissipative generation of •OH, and HRP‐activity toward exogenously added H_2_O_2_. The cascade catalytic generation of •OH was limited by the electron storage capacity of semiquinone radicals, which led to a collaboratively enhanced and gradually attenuated ROS generation by coupling photodynamic modules. Moreover, PDANFs with 1D morphology could fully use H_2_O_2_ in TME to continuously catalyze the generation of low doses of •OH for sustained immunomodulation. Furthermore, the HRP‐like catalytic activity of PDANFs was strongly pH‐dependent, with the catalytic efficiency of GH complexes significantly higher (2.6‐fold) in acidic pH conditions (pH 6.5) than in neutral pH conditions (pH 7.4) (Figure S13). These results indicated that ROS generation is selectively triggered by NIR irradiation in the pathologically acidic TME, minimizing potential effects on normal tissues under physiological pH and without NIR irradiation.

### 2.3 Cytotoxicity and Immune Activation Effect

**FIGURE 4:**
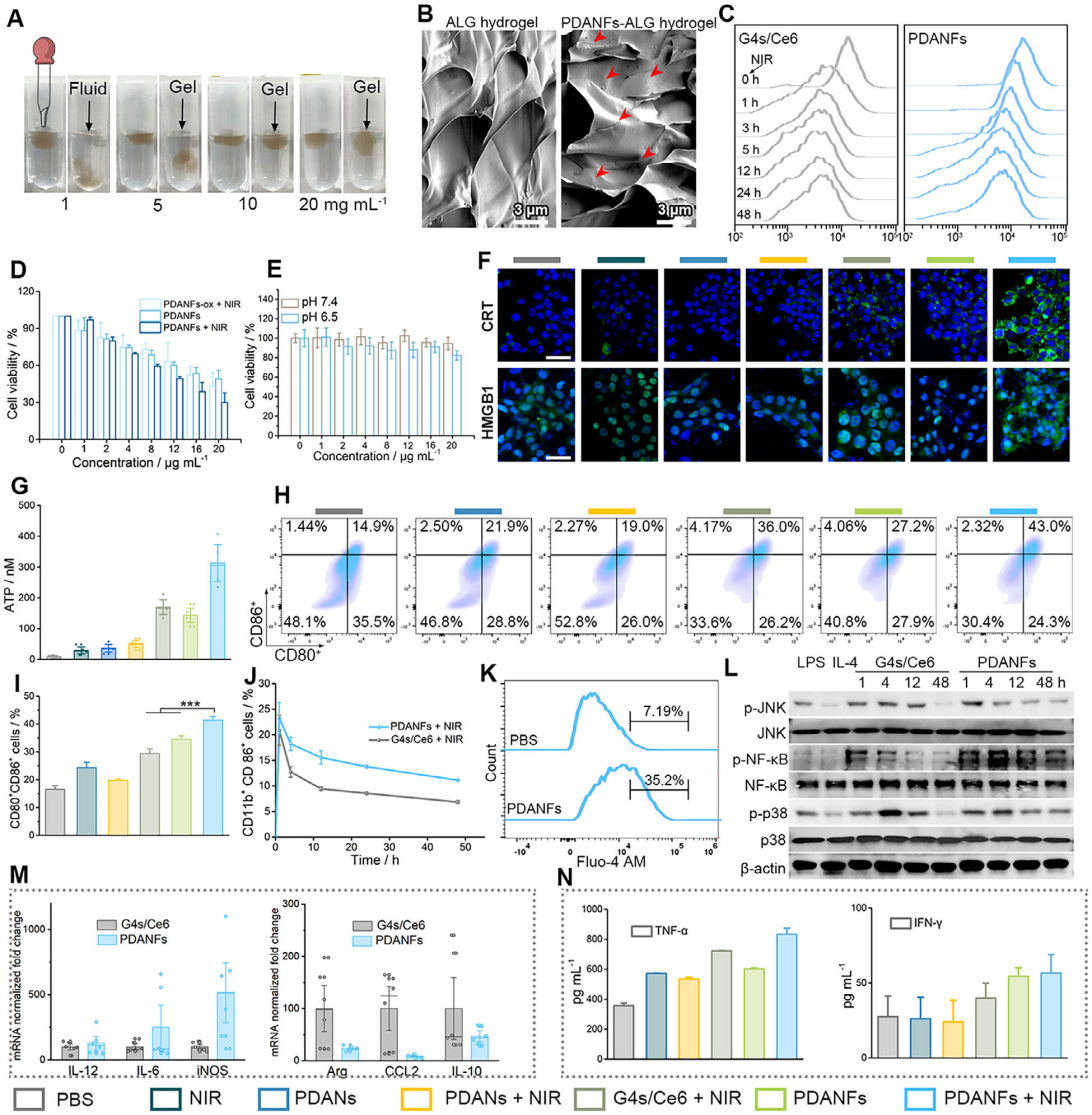
In vitro cancer cell inhibition and TIM remodeling. (A) Photographs showing the gelation behavior of PDANFs‐ALG‐hydrogel at different ALG concentrations (1, 5, 10, and 20 µg mL^−1^). (B) SEM images of the ALG hydrogel and PDANFs‐ALG hydrogel. The red arrow indicates uniform distribution of PDANFs (C). Flow cytometry analysis of intracellular ROS levels of 4T1 cells treated with G4s/Ce6‐ALG hydrogel and PDANFs‐ALG hydrogel plus NIR irradiation. (D) Cell viability of 4T1 with different treatments under acidic pH conditions. (E) Cell viability of 4T1 incubated with PDANFs‐ox‐ALG hydrogel (PDANFs‐ox concentration: 20 µg mL^−1^) under different pH conditions. (F) Immunofluorescence analysis of CRT and HMGB1 in cells after the different treatments. Scale bar: 40 µm. (G) ATP secretion after the various treatments. (H, I) Flow cytometry images and quantitative analysis of CD11c^+^ CD80^+^ CD86^+^ BMDCs after co‐incubation with 4T1. (J) Time‐dependent quantitative changes of CD11b^+^ CD86^+^ BMMs after co‐incubation with 4T1. (K) Quantitative analysis of calcium levels in BMMs with different treatments. (L) Immunoblot of BMMs treated with G4s/Ce6 or PDANFs plus 660 nm laser illumination. Examination of the expression of M1 and M2 markers in BMMs by ELISA (M) and RT‐qPCR (N).

Given that sustained ROS generation was time‐consuming, the retention of the nanocatalyst at tumor sites needed to be extended. Therefore, PDANFs were encapsulated in alginate (ALG) hydrogel for subsequent cellular and in vivo experiments. ALG is used as a water‐soluble natural polysaccharide that can be crosslinked by Ca^2+^ at physiological concentration [36]. It was found that 1 mg mL^−1^ of ALG solution injected into the Ca^2+^‐containing PBS showed fluid‐like behavior, whereas ALG at higher concentrations (5 mg mL^−1^ and above) rapidly transformed into gels (Figure 4A). In addition, the doping of PDANFs had negligible effects on the pore features of hydrogels (Figure 4B). Moreover, the impact of hydrogel encapsulation on the ROS generation efficiency of PDANFs was evaluated by employing DCFH as a total ROS probe. As shown in Figure S14, owing to the porous structure of the hydrogel, it exerted a negligible influence on ROS production.

Next, the dynamics of ROS generation were evaluated by confocal laser scanning microscopy (CLSM) and flow cytometry. As shown in Figure 4C, Figures S15 and S16, 4T1 cells treated with G4s/Ce6‐ALG‐hydrogel followed by NIR irradiation showed a strong initial ROS signal, which decreased markedly (∼84% of reduction) within 1 h. In contrast, upon treatment with PDANFs‐ALG‐hydrogel, the initial ROS signal showed a 1.45‐fold higher level than that in the G4s/Ce6‐ALG‐hydrogel group, reflecting the synergistic contribution from multiple ROS sources. The signal then slowly decreased, showing ∼53% and ∼66% reductions at 1 h and 3 h, respectively. Notably, after 48 h of treatment, the PDANFs‐ALG‐hydrogel‐treated group maintained ∼1.42‐fold higher ROS signal than the G4s/Ce6‐ALG‐hydrogel group, demonstrating its superior and sustained ROS generation capability.

Considering the explosive ^1^O_2_ generation by photodynamic effect and dissipative •OH generation through nanoconfined cascade reaction could prolong the generation of ROS with an effective therapeutic threshold level and improve the immune activation, the antitumor efficacy of PDANFs was first evaluated in vitro using 4T1 cells under pathological acidic environment (pH 6.5). Upon NIR irradiation, the cell viability was decreased to ∼43% at 20 µg mL^−1^ of PDANFs‐ox. Upon using PDANFs but in a dark condition, the cell viability also decreased in a concentration‐dependent manner, decreased to ∼49% at the highest concentration of 20 µg mL^−1^. The results demonstrated that both explosive ^1^O_2_ and the dissipative •OH effectively suppressed tumor cell growth. Moreover, upon the cells treated with PDANFs plus NIR irradiation, the cell viability continuously decreased to 30%, suggesting a strong synergistic cytotoxic effect from the combined explosive ^1^O_2_ and the dissipative •OH generation (Figure 4D).

Additionally, the cell viability of 4T1 remained above 95% when treated with PDANFs‐ox in neutral pH conditions (Figure 4E). Under acidic conditions (pH 6.5), only ∼20% reduction in cell viability was observed at PDANFs‐ox concentrations above 20 µg mL^−^^1^, suggesting that the pathological H_2_O_2_‐dependent HRP‐like activity of PDANFs induced minimal cytotoxicity. Thus, these results indicated that the cytotoxicity of PDANFs was mainly contributed by the adequate ROS generation from the photodynamic process and cascade catalytic reaction. Moreover, PDANFs exhibited a negligible inhibitory effect against L929 cells under a neutral pH environment (pH 7.4), which indicated the good biocompatibility of PDANFs (Figure S17). Furthermore, the therapeutic effect of PDANFs was evaluated by live and dead staining and CCK‐8 assays. As shown in Figures S18 and S19, PDANFs induced markedly higher cytotoxicity compared to PDANs, highlighting the important role of semiquinone and catalytic cascade reaction in cytotoxicity. Besides, G4s/Ce6 + NIR achieved only 55% inhibition of cell growth, which was lower than the PDANFs + NIR group due to the transient ROS generation.

To explore the mechanism of tumor cell death induced by PDANFs, we examined morphological changes by microscopy. As shown in Figure S20A, cells treated with G4s/Ce6 or PDANFs plus NIR irradiation showed pronounced swelling and membrane bursting with bubbles, which was more severe in the PDANFs group, consistent with higher ROS production. Because the cell membrane is critical for cellular integrity, oxidative damage to the membrane would destroy the cell integrity to release cellular contents, resembling pyroptosis. GSDMD and Caspase‐3 proteins were analyzed by western blotting. GSDMD could be specifically cleaved by Cleaved‐Caspase‐3 to release gasdermin D‐N (GSDMD‐N), which forms membrane pores and induces pyroptosis. As shown in Figure S20B–D, GSDMD‐N and Cleaved‐Caspase‐3 levels were considerably enhanced in the groups of G4s/Ce6+NIR and PDANFs+NIR, especially the latter. Furthermore, the flow cytometry data showed that the PDANFs group had the highest proportion of Annexin V‐FITC+/7AAD+ cells (52.1%, primarily pyroptosis) (Figure S21), implying that extracellular ROS mainly caused cell death via pyroptosis.

Subsequent exploration was undertaken to investigate the ICD activation, with a primary focus on calreticulin (CRT) exposed on the cell surface, high mobility group box 1 (HMGB1) secreted by tumor cells, and ATP molecules released by cells. As shown in Figure 4F, PDANFs + NIR‐treated cells showed a stronger increase in CRT exposure in the cytoplasm than the Ce6/G4s + NIR group and the PDANFs group. The results were also confirmed by the decrease of HMGB1 level within the nucleus and the elevated leakage of HMGB1 and ATP from cancer cells (Figure 4F,G, and Figure S22). Together, these results indicated that prolonging ROS generation duration with effective cytotoxic concentrations induced adequate ICD, laying a foundation for sufficient immune activation.

During the ICD process, dead cells release residues that serve as tumor antigens for dendritic cells (DCs) to capture in the tumor environment. As the principal antigen‐presenting cells (APCs), DCs internalize and process these antigens, then migrate to nearby draining lymph nodes, where they mature and initiate adaptive immune responses. A co‐culture system composed of mouse bone marrow‐derived dendritic cells (BMDCs) and 4T1 cells was established to investigate the immune activation triggered by the ROS nanogenerator (Figure S23). Specifically, 4T1 cells were pretreated with nanomaterial‐ALG hydrogel and NIR irradiation. Then, the pretreated 4T1 cells and immature BMDCs were co‐cultured for 24 h. Flow‐cytometric analysis showed that PDANFs + NIR group induced a markedly higher BMDCs maturation than the G4s/Ce6 + NIR group (1.2‐fold) and the PDANFs group (1.6‐fold) (Figure 4H,I).

It has been reported that DAMPs and ROS stimulation could reprogram the TAMs from M2 phenotype to M1 phenotype to remodel the tumor immune‐microenvironment [37]. However, the repolarized M1 phenotype could belatedly return to the M2 phenotype. Therefore, strategies capable of achieving long‐term M2‐to‐M1 repolarization and maintain macrophages in an activated M1state are important. Owing to the ability of PDANFs to catalytically convert endogenous H_2_O_2_ within the tumor microenvironment into ROS in a sustained manner, these nanofibers hold promise for the prolonged maintenance of the antitumor phenotype of macrophages. First, the ROS concentration threshold required for sustaining the M1 phenotype without cytotoxic effects was explored. As shown in Figure S24, as the concentration of H_2_O_2_ increased, the polarization efficiency of BMMs initially rose. However, when the H_2_O_2_ concentration exceeded 150 µM, the polarization efficiency exhibited a declining trend. Concurrently, an increase in apoptosis levels was observed. This phenomenon indicated that excessive H_2_O_2_, following conversion to ROS by PDANFs, primarily exerted cytotoxic effects rather than serving as a pro‐polarization stimulus (Figure S25). According to previous reports, the H_2_O_2_ concentration in the tumor microenvironment ranges from 50 to 100 µM, which enables the nanofibers to achieve effective polarization of macrophages [38].

To further verify this phenomenon, a transwell co‐culture system of mouse bone marrow‐derived macrophages (BMMs) and 4T1 was used to verify the maintenance of M1 phenotype (Figure S23). Results showed that PDANFs‐treated group showed a slower downtrend and a higher amount of M1 macrophages (marketed by CD86) than the G4s/Ce6 group after 4 h (1.4‐fold) and 48 h (1.6‐fold) of treatment, respectively (Figure 4J). Moreover, the mechanism of M2 macrophage repolarization by DAMPs and ROS stimulation was then investigated. It has been reported that extracellular ROS could open the Transient Receptor Potential (TRP) channels, leading to an elevation of cytosolic Ca^2+^. The increased intracellular Ca^2+^ contributes to activation of NADPH oxidase, resulting in increased intracellular ROS, which activate the MAPKs/NF‐κB signaling [39, 40]. The intracellular calcium levels were detected with a calcium‐sensitive fluorescent dye, Fluo‐4 AM, and found that the signal was significantly increased in the PDANF groups (Figure 4K). Moreover, signaling activation of BMMs after being treated with PDANFs or G4s/Ce6 was analyzed. The LPS treatment groups and IL‐4 treatment groups were set up as positive and negative controls, respectively. Substantial increase in phosphorylation of JNK, NF‐κB, and p38 was observed as early as 1 h after co‐culturing M2 macrophage with PDANFs plus NIR irradiation and sustained for up to 48 h, which was delayed and not significant until 12 h after G4s/Ce6 plus NIR treatment (Figure 4L and Figure S26).

The repolarization effect of PDANFs was also confirmed by the down‐expression of anti‐inflammatory signature genes (Arg, CCL‐2, and IL‐10), and the up‐expression of inflammatory signature genes (IL‐12, IL‐6, and iNOS) (Figure 4M). Besides, the related cytokines secreted by BMMs were further detected. A significant upregulation of pro‐inflammatory cytokines, including TNF‐α and IFN‐γ, secreted by BMMs was observed (Figure 4N). These results collectively illustrated that the DAMPs and ROS could effectively repolarize M2 macrophages, and the activation of M1 signaling by stepped‐persistent ROS generation was more effective and sustained compared with that induced by transient ROS generation.

### 2.4 PDANFs‐Based ROS Nanogenerators for Tumor Inhibition

**FIGURE 5:**
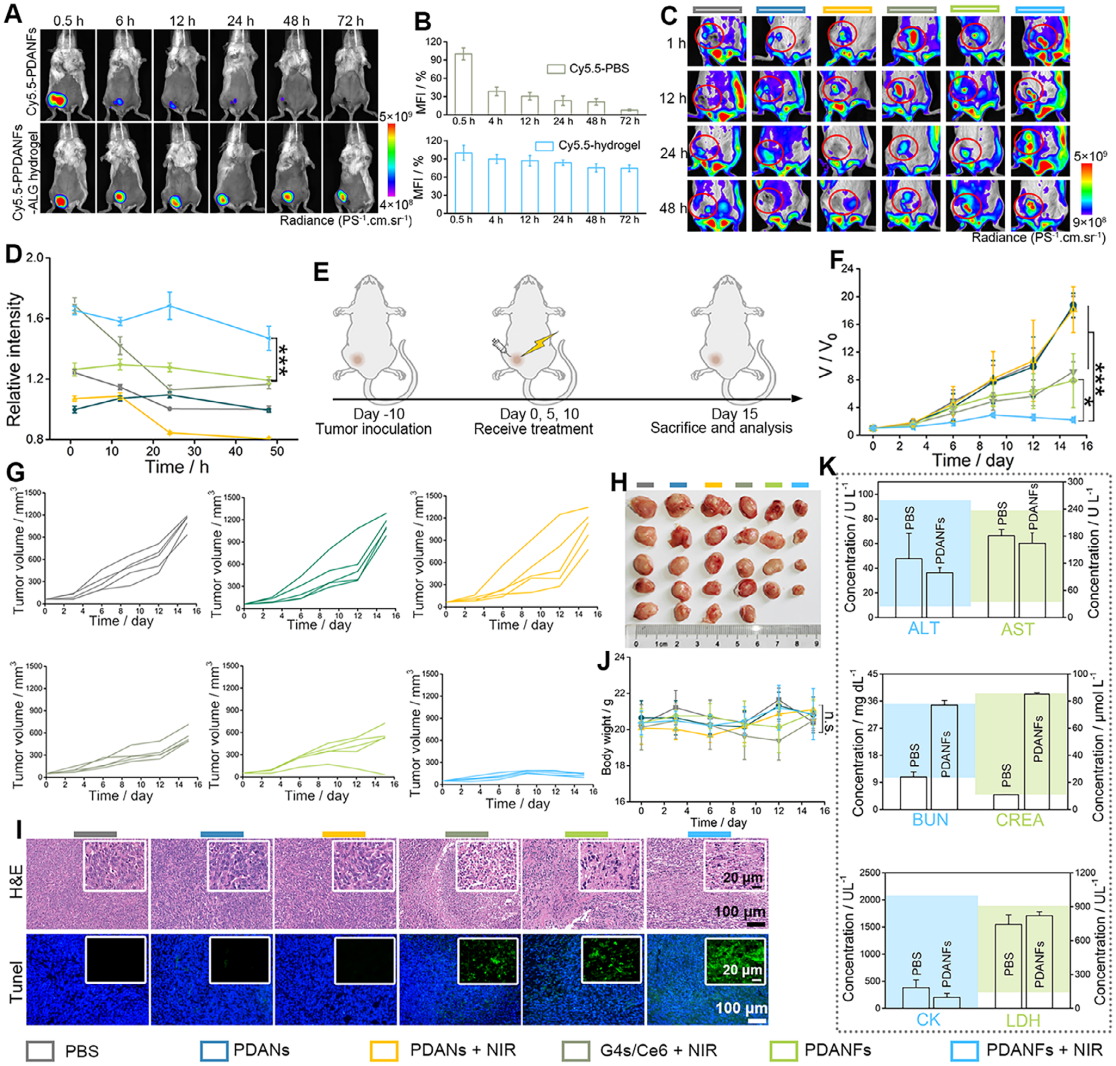
PDANFs‐based ROS nanogenerators for in vivo tumor therapy. (A) In vivo fluorescence images of tumor‐bearing mice at different time points after intratumoral injection of Cy5.5‐PDANFs or Cy5.5‐PDANFs/ALG hydrogel. (B) Corresponding mean fluorescence intensity (MFI) at the tumor sites in (A), with data normalized to the intensity at 0.5 h. (C,D) In vivo fluorescence images and quantitative results of ROS level at tumor sites with different treatments. (E) The schematic establishment and treatment of the 4T1 tumor‐bearing mice model. (F) Relative tumor volume from 0 to 15 days after various treatments. (G) Individual tumor growth data for each treatment group. (H) Digital photos of the dissected tumors from each group. (I) H&E staining and TUNEL images of tumor tissues on the 15th day. (J) Body weight changes of tumor‐bearing mice during treatment. (K) Liver function, renal function, and heart function. ALT, alanine aminotransferase; AST, aspartate transaminase; BUN, Blood urea nitrogen; CREA, creatinine; CK, creatine kinase; LDH, lactic dehydrogenase. The blue and green‐filled areas indicate the normal range of the biochemical indicators. Error bars are based on the standard error of the mean (*n* = 5). ^**^ *p* < 0.01, ^***^ *p* < 0.001, and *n.s*. represents no significant difference determined by Student's *t*‐test.

Before verification of the in vivo antitumor effect of PDANFs, the gelation behavior of ALG was evaluated by intratumorally injecting Cy5.5‐labeled PDANFs encapsulated in ALG hybrid fluids at various ALG concentrations (0, 1, 5, 10, and 20 mg mL^−1^). Cy5.5‐PDANFs encapsulated in low‐concentration ALG hydrogel (1 mg mL^−^^1^) exhibited minimal tumor retention at 72 h post‐injection, comparable to that of free Cy5.5‐ PDANFs (Figure 5A,B, and Figure S27). While Cy5.5‐PDANFs formulated with higher ALG concentrations (≥10 mg mL^−^^1^) showed strong tumor localization, with approximately 75% of the mean fluorescence intensity (MFI) retained after 72 h. Moreover, cryosectioning of tumor tissues revealed uniform distribution of the hydrogel within the tumor tissue (Figure S28). Thus, an ALG concentration of 10 mg mL^−1^ was determined to be optimal, providing a relatively homogeneous intratumoral distribution and effective tumor retention.

Dynamic fluorescence imaging was performed to monitor in vivo ROS generation under different treatment conditions (Figure 5C,D). Control groups exhibited weak fluorescence signals consistent with the low ROS content in tumor tissues. PDANs + NIR group showed a slight signal increase due to heat stress‐induced free radicals. In contrast, ROS was explosively generated in the G4s/Ce6 + NIR group, which decreased rapidly after 12 h of administration. Moreover, the signals showed a slight increase and were maintained at a relatively high level in the PDANFs group compared to the G4s/Ce6 + NIR group. Furthermore, PDANFs + NIR group showed an initial explosive increase in ROS signals, followed by a slowly decreased signal, and also maintained at a relatively high level (1.3‐fold) than that of the G4s/Ce6 + NIR group even after 48 h. The results above demonstrated that PDANFs have successfully integrated different ROS generation modules and showed a stepwise and sustained ROS generation dynamic in vivo.

To observe the effect of tumor hypoxic microenvironment on ROS production, tumor models with varying sizes were established. Immunohistochemical analysis of HIF‐1α revealed markedly higher hypoxic levels in larger tumors (Figure S29A). Besides, ROS generation was reduced in larger tumor tissues, due to the limited oxygen availability under hypoxic conditions (Figure S29B,C).

Next, the antitumor effects were evaluated (Figure 5E). Tumor growth curve and the extracted tumors after 15 days of treatment were displayed in Figure 5F–H. Compared with the PBS group, the PDANs group showed a negligible therapeutic effect due to the low semiquinone radical content for ROS generation. Besides, PDANs + NIR group also showed negligible tumor, indicating that the therapeutic effect primarily originated from ROS generation rather than the mild photothermal effect during illumination (Figure S30). Moreover, G4s/Ce6 + NIR and PDANFs treatments induced tumor inhibition rates of 51% and 58%, respectively, whereas PDANFs + NIR treatment produced a markedly higher inhibition of 88%. Hematoxylin and eosin (H&E) staining of tumor tissues showed that more condensed nuclei and typical histopathological damage of cancer cells in the PDANFs + NIR group compared to other groups (Figure 5I). Consistently, terminal deoxynucleotidyl transferase‐mediated dUTP nick end labeling (TUNEL) staining also showed the strongest positive signals in PDANFs + NIR group (Figure 5I). Additionally, no significant changes in body weight were observed across treatment groups (Figure 5J). H&E staining of major organs (liver, heart, kidney, spleen, and lung) (Figure S31) and blood biochemical analyses (Figure 5K and Figure S32) indicated good biocompatibility. Collectively, these results demonstrate that PDANFs successfully integrate multiple ROS generation modules, enabling both rapid and sustained ROS production and achieving superior antitumor efficacy with favorable safety.

### 2.5 Stepped‐Persistent ROS Generation for Sustained Immunoactivation

**FIGURE 6:**
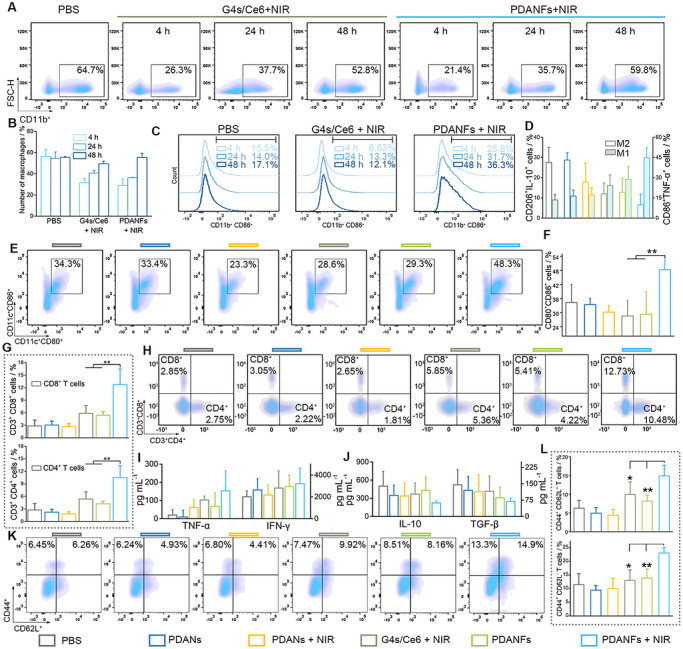
Immune stimulation effect of PDANFs in vivo. (A,B) Representative flow cytometric analysis images and quantitative results of macrophage (CD11b^+^) number at 4, 24, and 48 h after G4s/Ce6 + NIR treatment or PDANFs + NIR treatment. (C) Representative flow cytometric analysis images of macrophage (CD11b^+^ CD86^+^) number at 4, 24, and 48 h after G4s/Ce6 + NIR treatment or PDANFs + NIR treatment. (D) Quantitative analysis of M1 (CD86^+^ TNF‐α^+^) and M2 (CD206^+^ IL‐10^+^) in tumor tissues after 15 days of different treatments. (E,F) Representative flow cytometric analysis images and quantitative results of mature DCs (CD11c^+^ CD80^+^CD86^+^) in tumor tissues after different treatments. (G,H) Representative flow cytometric analysis images and quantitative results of CD4^+^ T cells and CD8^+^ T cells in tumor tissues after mice with different treatments. (I,J) The levels of TNF‐α, IFN‐γ, IL‐10, and TGF‐β expression in tumor tissues after mice with different treatments. (K,L) Flow cytometric images and quantitative analysis of central memory T cells (T_CM_, CD44^+^ CD62L^+^ CD8^+^) and effector memory T cells (T_EM_, CD44^+^ CD62L^−^ CD8^+^) in spleen after different treatments (*n* = 5). ^*^ *p* < 0.05, ^**^ *p* < 0.01 determined by Student's *t*‐test.

To investigate the antitumor immune mechanisms underlying the superior antitumor efficacy of PDANFs, flow cytometry analysis was performed. The total number of macrophages initially decreased and then returned to the nontreatment levels following treatment with G4s/Ce6 or PDANFs plus illumination (Figure 6A,B). These data suggested that ROS indiscriminately damaged both tumor cells and TAMs, while tumor‐derived DAMPs subsequently recruited new macrophages into the tumor tissue. Notably, the proportion of CD11b^+^CD86^+^ cells (M1 macrophages) remained at a low level over time after G4s/Ce6+ NIR treatment, whereas it continuously increased in the PDANFs + NIR group (Figure 6C). Moreover, tumor tissues collected from mice 15 days after different treatments were further analyzed (Figure 6D and Figure S33). Compared with other groups, PDANFs + NIR treatment not only markedly increased the proportion of M1 (CD86^+^ TNF‐α^+^), but also reduced M2 (CD206^+^ IL‐10^+^) cells in tumor tissues. This repolarization effect could be attributed to the effective elimination of original TAMs, as well as the sustained activation of the recruited macrophages.

Considering the massive ROS generation could induce ICD process and active adaptive immune response, tumor‐draining lymph nodes of mice were analyzed by flow cytometry to assess DCs maturation (Figure 6E,F). The percentage of mature DCs (CD11c^+^ CD80^+^CD86^+^) was markedly higher in the PDANFs + NIR group than in other groups. Flow cytometry of tumor tissues revealed that the intratumoral infiltration of CD3^+^CD8^+^ T cells (cytotoxic T cells) and CD3^+^CD4^+^ T cells (helper T cells in adaptive immunity) was markedly higher in the PDANFs + NIR group than in other groups (Figure 6G,H). Consistently, ELISA of tumor lysates further showed a notable increase in antitumor cytokines (TNF‐α, IFN‐γ) and a decrease in immunosuppressive cytokines (IL‐10, TGF‐β) (Figure 6I,J). These data suggest that the triggered ICD facilitated cytotoxic T‐cell recruitment and activation.

To further investigate local treatment, the stepwise‐persistent ROS nanogenerator could elicit not only local immune responses, but also a systemic immune response. A bilateral tumor model was used to mimic tumor metastasis (Figure S34A). PDANFs + NIR treatment significantly inhibited the growth of both primary and distant tumors, compared to other groups (Figure S34B,C). Specifically, distal tumors in the PBS group, PDANs group, and PDANs + NIR group grew rapidly, with an average volume ranging from 60 to 750–850 mm^3^ (12.5–14.2‐fold increased) after 15 days of intratumoral administration, whereas the tumors grew only 5–7 folds (300–420 mm^3^) in the PDANFs group and G4s/Ce6 + NIR group. Extraordinarily, the tumor volume remained only 30 mm^3^ in the PDANFs + NIR group. Immunofluorescence staining of distant tumors displayed strong CD4^+^ and CD8^+^ T‐cell infiltration, confirming that PDANFs + NIR induced potent systemic antitumor immunity (Figure S35).

Based on this strong systemic immune response, we further investigated whether PDANFs + NIR could inhibit tumor metastasis. After intravenous injection of 4T1 cells, mice receiving PDANFs + NIR showed the most pronounced inhibition of lung metastases, which was also confirmed by H&E analyses (Figure S36). Moreover, flow cytometry revealed significantly higher percentages of effector memory T cells (T_EM_, CD3^+^CD8^+^CD62L^−^CD44^+^) and central memory T cells (T_CM_, CD3^+^CD8^+^CD62L^+^CD44^+^) within the spleen, indicating durable immunological memory and long‐term protection (Figure 6K,L).

**FIGURE 7:**
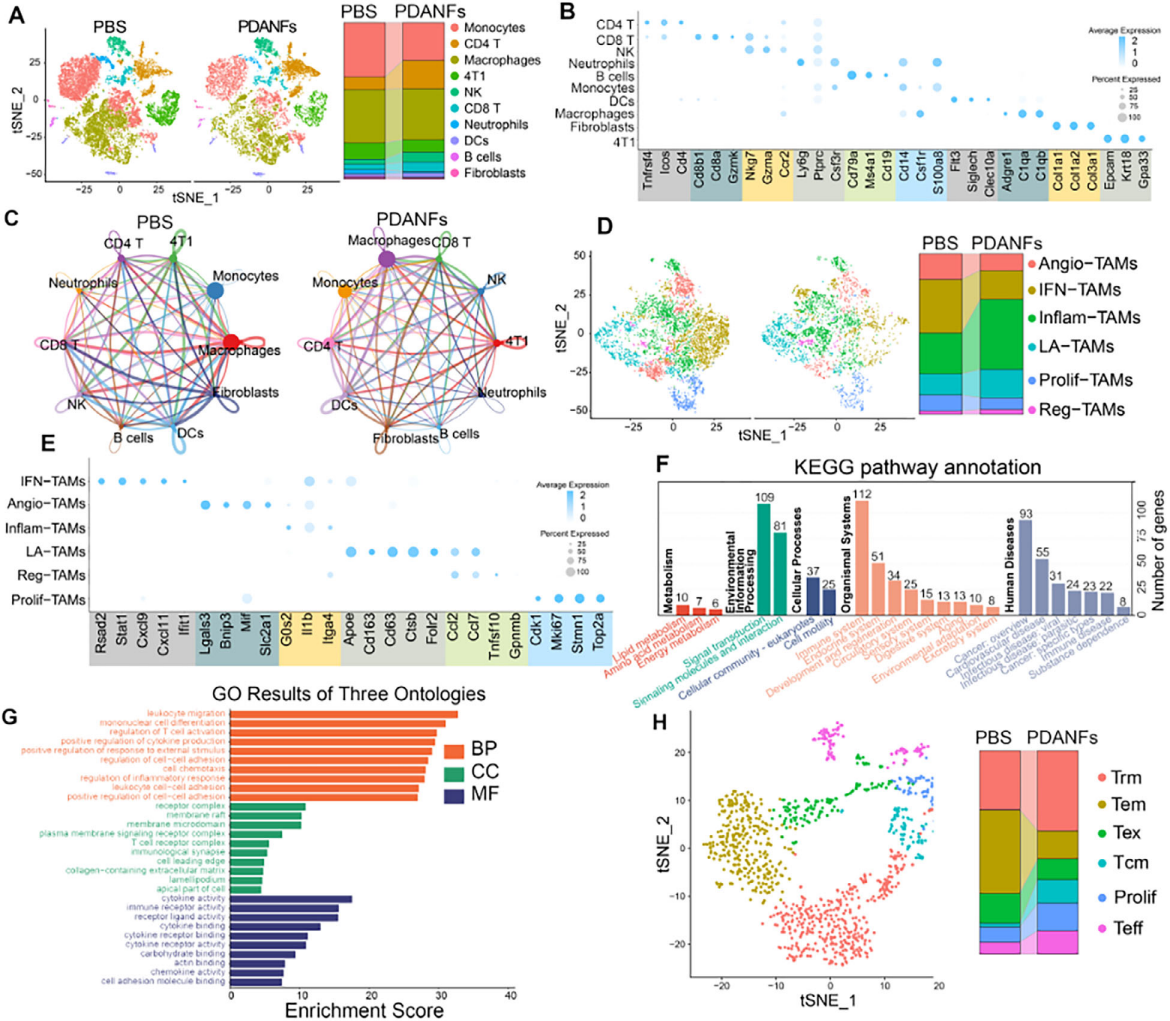
Single‐cell RNA sequencing analysis of the reprogramming effect of PDANFs on 4T1 tumor immune cell population. (A) T‐distributed stochastic neighbor embedding (t‐SNE) visualization of clustering of representative markers of cells from the tumor, each dot corresponds to one single cell. (B) Dot plots showing normalized expression of selected marker genes for the main lineages. The color represents the mean expression level, and the size indicates the proportions of cells expressing the genes. (C) The overall network of cell‐cell communication among multiple immune cell types. (D) t‐SNE visualization of the macrophages. (E) Dot plots showing representative top marker genes across the macrophage subtypes. (F) KEGG pathway enrichment analysis of differentially expressed genes of macrophages in the PDANFs group compared to the PBS group. (G) The top‐ranking terms in gene ontology analyses of biological processes (BP), cellular components (CC), and molecular functions (MF) of macrophages in the PDANFs group compared to the PBS group. (H) t‐SNE mapping and relative proportions of different T cell subtypes in the PBS group and the PDANFs group.

To evaluate the TME reprogramming effects of PDANFs + NIR, single‐cell RNA (scRNA) sequencing was employed to analyze immune cell populations within 4T1 tumors. Through unsupervised clustering, a total of 42 cell clusters and 10 major cell types were classified using established cell markers (Figure 7A,B). PDANF treatment reduced the proportion of 4T1 tumor cells and obviously increased the interaction of macrophages with other cell types (Figure 7A–C and Figure S37A). Sub‐clustering analysis of macrophage identified into seven subtypes according to the literature [41, 42] (Figure 7D,E, and Figure S37B). Three subtypes of macrophages exhibited significant proportional changes after PDANFs treatment, including IFN‐TAMs, Angio‐TAMs, and Inflam‐TAMs. IFN‐TAM, although M1‐like in appearance, resembles recently identified immunosuppressive macrophages that suppress immune responses through tryptophan degradation and the recruitment of immunosuppressive regulatory T cells (Tregs) [41]. Angio‐TAMs, characterized by the high expression of angiogenic signatures, are associated with tumor progression and poor prognosis. Inflam‐TAMs express inflammatory cytokines and actively recruit and regulate immune cells during the tumor‐associated inflammatory response. PDANFs treatment decreased IFN‐TAMs and Angio‐TAMs, while increasing Inflam‐TAMs, indicating successful tumor immune microenvironment reprogramming.

Subsequently, we conducted pathway analyses and found that macrophages in the PDANFs group were enriched in signal transduction and the immune system (Figure 7F). Consistently, Gene ontology (GO) analysis revealed that PDANFs treatment positively correlated with immune activation and cytokine production (Figure 7G). Moreover, PDANFs treatment markedly increased the proportion of tumor‐infiltration DCs, CD4^+^ T cells, and CD8^+^ T cells (Figure 7A). Further annotation of CD8^+^ T cells into six subpopulations showed a notable increase in effector CD8^+^ T cells (Teff) and tissue‐resident memory T cells (Trm) (Figure 7H and Figure S37C). Collectively, these findings indicate that PDANFs treatment reprogrammed immunosuppressive macrophages and activated CD8^+^ T cell anti‐tumor immune responses.

## 3 Conclusion

In summary, a type of stepped‐persistent ROS nanogenerator (PDANFs) was successfully synthesized for enhancing antitumor immunity. In this design, G4s‐containing DNA nanochains loaded with hemin and Ce6 acted as a supramolecular host for stabilizing semiquinone radicals during PDA polymerization and acted as a structure‐directing agent to synthesize PDA nanofibers. In TME, the nanofibers explosively generate ^1^O_2_ under NIR irradiation, and dissipatively generate •OH through a nanoconfined cascade reaction between semiquinone radicals and G4s/hemin DNAzyme, as well as continuously catalyze the H_2_O_2_ in TME to generate •OH. Both in vitro and in vivo experiments verified that PDANFs enabled rapid and sustained ROS production, maintained macrophages in the antitumor M1 phenotype, thereby promoted robust antitumor immunity, and induced durable immunological memory, achieving superior therapeutic efficacy in multiple mouse tumor models with excellent safety. Therefore, this strategy not only provided a new and general method for interface‐oriented synthesis of ROS nanogenerators but also offered a promising platform for controllable ROS‐based immunotherapy.

## 4 Materials and Methods

### 4.1 Synthesis of PDANFs

To prepare G‐quadruplexes (G4s)‐containing nanowires, toehold strand, H1, H2, with a molar ratio of 1:200:200, were added into an annealing buffer (10 mM Tris pH 7.5, 1 mM EDTA, 50 mM MgCl_2_), and mixed uniformly at room temperature. The self‐assembled products were purified with the SanPrep Column PCR Product Purification Kit. To prepare the G4 nanowire/Ce6/Hemin complexes, Ce6 and Hemin per‐dispersed in DMSO were added to the G4s nanowires solution (molar ratio, Ce6/Hemin/H1/H2 = 3:3:1:1).

For the synthesis of PDANFs, G4s nanowires/Ce6/Hemin complexes and dopamine hydrochloride (1 mg) were added into 1 mL of Tris (2.5 mg) solution and stirred for 24 h at room temperature. The weight ratio of DNA/dopamine ranged from 0.03125:1 to 1:1. The final products were separated by centrifugation.

### 4.2 Intracellular ROS Detection

4T1 cells were seeded onto confocal dishes for 12 h and then treated with G4s/Ce6‐ALG and PDANFs‐ALG, followed by NIR irradiation. The cells at different treatment time points were stained with DCFH‐DA (10 µM, 30 min). Then, the fluorescence signals of the cells were evaluated with a CLSM and flow cytometry.

### 4.3 In Vitro Inhibition of Cancer Cells

First, PDANs (20 µg mL^−1^), G4s nanowires/Ce6 complexes (4 µg mL^−1^), and PDANFs (20 µg mL^−1^) containing ALG‐hydrogel (10 mg mL^−1^) were coated on the 24‐well plates. Then, 4T1 cells were planted into the hydrogel‐precoated 24‐well plates at a density of 5 × 10^4^ cells per well and cultured at hypoxic and neutral pH conditions (1% O_2_, pH 7.4) for 12 h. Then, the cells with or without NIR treatment were further incubated at normoxia and pathologically pH condition (21% O_2_, pH 6.5) for 24 h. Calcein‐AM‐PI staining reagents were applied to stain the viable cells as green fluorescence (λ_ex_ = 490 nm, λ_em_ = 515 nm) and dead cells as red fluorescence (λ_ex_ = 535 nm, λ_em_ = 617 nm). After 30 min of incubation, the staining solution was removed and rinsed with PBS twice, and the samples were subsequently visualized by CLSM. The cell viability was measured using the CCK‐8 assay. Briefly, after the cells were incubated with different materials for 24 h, the medium was removed, and the wells were washed with PBS. Subsequently, 20 µL of CCK‐8 together with 200 µL of fresh medium was added, and the cells were further incubated for 3 h. Finally, the absorbance of each well at 450 nm was measured using a microplate reader (Model 680, Bio‐Rad, USA).

### 4.4 ICD Analysis

4T1 cells with different treatments were fixed with 4% paraformaldehyde for 10 min and then submerged in immunostaining blocking solution at 4°C for 12 h. For CRT analysis, the cells were incubated with a CRT rabbit monoclonal antibody (1:200) at 4°C overnight. Then, 1 mL of Alexa Fluor 488‐conjugated goat anti‐rabbit IgG (1:300) was added, and the cells were further incubated for 1 h. Nuclear were counterstained with DAPI at room temperature for 10 min. Fluorescence was detected under a confocal microscope. HMGB‐1 and ATP assays were conducted following the manufacturer's instructions.

### 4.5 In Vitro Macrophage Polarization and DC Activation

To investigate the ROS‐induced polarization of macrophages, a co‐culture model of 4T1 and BMMs was established. Briefly, 4T1 cells (2.5 × 10^4^ per well) were seeded onto nanomaterial‐hydrogel coated 24‐well plates and cultured at hypoxic and neutral pH condition (1% O_2_, pH 7.4) for 12 h. Besides, BMMs were incubated in the upper layer of the transwell chamber (2.5 × 10^4^ per well) and cultured in a new 24‐well plate for 12 h. After the 4T1 cells were treated with NIR irradiation, BMMs were co‐cultured with 4T1 cells at normoxic and acidic pH condition (21% O_2_, pH 6.5). After incubating for different times, BMMs were collected for apoptosis analysis, WB analysis, RT‐PCR analysis, and flow cytometric analysis. Intracellular calcium was measured using the calcium‐sensitive probe Fluo‐4 AM (Invirtrogen, USA) according to the manufacturer's instructions. Moreover, the medium samples were collected and analyzed by ELISA kits.

To investigate the DC activation, a co‐culture model of 4T1 and BMDC was established. Briefly, 4T1 cells (2.5 × 10^4^ per well) were seeded onto nanomaterial‐hydrogel‐coated 24‐well plates and cultured at hypoxic and neutral pH conditions (1% O_2_, pH 7.4) for 12 h. Besides, BMMs were incubated in the upper layer of the transwell chamber (2.5 × 10^4^ per well) and cultured in a new 24‐well plate for 12 h. After the 4T1 cells were treated with NIR irradiation, BMMs were co‐cultured with 4T1 cells at mormoxic and acidic pH condition (21% O_2_, pH 6.5). After incubating for different times, BMMs were collected for WB analysis, and the expression of CD206 and CD86 was detected by FCM. Moreover, the medium samples were collected and analyzed by ELISA kits.

### 4.6 Animals and Treatments

All animal experiments were approved by the Animal Ethics Committee of the Army Medical University. Six‐week‐old female BALB/c mice were purchased from Hunan SJA Laboratory Animal Co., Ltd., and fed for 1 week before the experiments. The subcutaneous tumor models were established by subcutaneous injection with 1 × 10^4^ 4T1 cells into the left mammary gland of each mouse. Then, tumor volume was calculated as (tumor length) × (tumor width)^2^ × 0.5. When the volume of the tumor reached 60–80 cm^3^, in vivo experiments were conducted.

### 4.7 In Vivo Gelation and Diffusion of PDANFs Hydrogel

In order to prepare fluorescent‐labeled PDANFs, PDANFs were first dispersed in Tris solution (pH 8.5). After adding Cy5.5‐PEG5000‐SH (w/w, PDANFs/Cy5.5‐PEG5000‐SH = 1:0.1) solution, the mixture was stirred at room temperature for 24 h. The excess Cy5.5‐PEG5000‐SH was removed by repeatedly washing the nanofibers with water three times. Thereafter, Cy5.5‐labeled PDANFs/ALG hybrid fluids with different ALG concentrations (0, 1, 5, 10, and 20 mg mL^−1^; 100 µL) were intratumor injected into tumor‐bearing mice. The body fluorescence imaging was recorded by an IVIS system.

### 4.8 In Vivo Anticancer Efficacy

Tumor‐bearing mice were randomly divided into six groups and defined as PBS hydrogel, PDANs hydrogel, PDANs hydrogel + NIR, G4s/Ce6 hydrogel + NIR, PDANFs hydrogel, and PDANFs hydrogel + NIR, respectively. The dosage of PDANFs was fixed at 100 mg kg^−1^, and the laser irradiation power density was 250 mW cm^−2^ (660 nm; 6 min). The administration was repeated every 5 days. At the end of the treatment, tumors and the major organs (heart, liver, spleen, lung, and kidney) were harvested for pathological analysis and immunological analysis. The sera were collected for serological analysis.

In the distant tumor model, 7 days after tumor cells were transplanted into the right mammary gland of the mice as a primary tumor, 4T1 cells were injected into the left mammary gland of each mouse to form the distant tumor. Three days later, different hydrogels were intratumorally injected into the primary tumor tissues, followed by NIR irradiation. The tumor size on each side was measured and quantified according to the above‐mentioned formula.

In the lung metastasis model of breast cancer, 7 days after 1 × 10^4^ 4T1 cells were transplanted into the left mammary gland of the mice, hydrogels were intratumorally injected, followed by NIR irradiation. The administration was repeated every 5 days. Moreover, 4T1 cells were injected into the tail vein on day 7. Mice were dissected after day 17, and lungs were removed, washed in PBS, and immediately placed in paraformaldehyde fixative.

### 4.9 In Vivo ROS Detection

After the tumor‐bearing mice received different treatments, five mice in each group were anesthetized by inhalation of isoflurane (2% in 100% oxygen). ROS probe DCFH was injected subcutaneously into the tumor tissues, and the body fluorescence imaging was recorded by an IVIS system.

### 4.10 Flow Cytometry and Cytokine Detection

To obtain single‐cell suspensions of tumors, tissues were mechanically dissected and incubated with 30 U/mL DAase, 175 U/mL Collagenase IV, and 100 U/mL HAse for 1 h, then filtered through a 75 µm filter. Similarly, tumor draining lymph nodes were incubated with 175 U/mL Collagenase IV for 1 h and then passed through a sieve to obtain lymph node single‐cell suspensions. Splenic tissues were passed through a sieve mesh and then treated with erythrocyte lysate to finally obtain a splenocyte single‐cell suspension. The cells were collected and washed twice with PBS before staining with fluorescence‐labeled antibodies.

Besides, tumor tissue was collected and incubated in RIPA buffer containing 1 mM PMSF and ground with grinding beads. The ground samples were centrifuged (12 000 g, 10 min), and the supernatant was collected. The supernatant was analyzed for levels of TNF‐α, IFN‐β, IL‐10, and TGF‐β using an ELISA kit according to the instructions.

### 4.11 Immunohistochemistry and Immunofluorescence Staining

Paraffin‐embedded slides of the tumor sample were dewaxed in xylene and rehydrated through 100%, 75%, and 50% ethanol, and then the antigen was repaired using sodium citrate. For immunohistochemical staining, slides were additionally treated with 3% hydrogen peroxide to eliminate endogenous peroxidase activity. After blocking with 5% goat serum, the slices were incubated with antibodies overnight at 4°C. The anti‐mouse/rabbit horseradish peroxidase‐labeled polymer (100 µL) was added, and the samples were incubated at 37°C for 30 min, followed by 100 µL of 3,3‐*N*‐diaminobenzidine tertrahydrochloride working solution and incubation at room temperature for 5 min. After 1 min of staining with hematoxylin, samples were washed in 50%, 75%, and 100% ethanol and xylene for 5 min, and neutral gum was used to seal the film. The film was observed and photographed under a microscope.

For immunofluorescence staining, slides were washed with PBS and stained with fluorephore‐conjugated secondary antibodies (1:500, *V/V*) at room temperature for 90 min and counter‐stained with 4’‐6‐diamidino‐2‐phenylindole for 2 min. After washing five times, the cells were observed with a confocal laser scanning microscope.

### 4.12 Single‐Cell RNA Sequencing

Single‐cell RNA sequencing of the tumor tissues of mice administered PDANFs was performed. Tumor tissues were enzymatically digested and processed into single‐cell suspensions. The oligo‐dT‐based complementary DNA database was barcoded using droplet segmentation via the Chromium Single‐cell Controller (10x Genomics) system in the NCI‐CCR single‐cell analysis tool. After removing dead cells, the cells were then diluted and loaded onto a NovaSeq 6000 (Illumina) for sequencing. Standard Seurat settings were employed for normalization and clustering.

### 4.13 Statistical Analysis

The results were presented as mean ± standard deviation (SD) (n ≥3). The statistical analysis was implemented with Origin 9.0 software through Student's *t*‐test, one‐way analysis of variance (ANOVA), or two‐way ANOVA. The statistical significances were indicated by ^*^ *p* < 0.05, ^**^ *p* < 0.01, and ^***^ *p* < 0.001.

## Conflicts of Interest

The authors declare no conflicts of interest.

## Supporting information




**Supporting File**: advs75713‐sup‐0001‐SuppMat.docx.

## Data Availability

The data that support the findings of this study are available on request from the corresponding author. The data are not publicly available due to privacy or ethical restrictions.
