# Supplementary material for: Supramolecular Assembly of Stepped‐Persistent ROS Nanogenerator for Sustained Tumor Immunotherapy
Source: Adv Sci (Weinh). 2026 May 15;13(45):e75713. doi: 10.1002/advs.75713 (PMC13335832; doi:10.1002/advs.75713)
Supplement: Supplementary file 1 [file ADVS-13-e75713-s001.docx]

Supporting Information

**Supramolecular Assembly of Stepped-Persistent ROS Nanogenerator for Sustained Tumor Immunotherapy**

Zhenqiang Wang, Ming Qin, Wenjing Lai, Shiwen Chen, Fengling Wang, Dandan Xie, Maohua Chen, Changpeng Hu, Kui Wang, Rong Zhang*, and Guobing Li*

**Affiliations**

Department of Pharmacy, The Second Affiliated Hospital, Third Military Medical University (Army Medical University), No. 83 Xinqiao Road, Chongqing 400037, China

***Corresponding authors.** Department of Pharmacy, The Second Affiliated Hospital, Third Military Medical University (Army Medical University), Chongqing 400037, China. Tel:+86-023-68774770.

Email: guobingl@tmmu.edu.cn (G. Li), zrcq73@tmmu.edu.cn (R. Zhang)

**Supplementary experimental section**

**Chemical and materials**

Unless otherwise noted, all reagent-grade chemicals were used as received, and distilled water was used to prepare all the aqueous solutions. DEPC (diethylpyrocarbonate) treated water, Hairpin 1 (GGG CGG GAT GGG ATG TAT GAT TGG TGA GAT ATG TAA TCA TAC ATC CGT TTC ATG GGT), Hairpin 2 (TGG GTA CAT ATC TCA CCA ATC ATA CAA TGA AAC GGA TGT ATG ATT GGG TGG GCG GGA), and Toehold (ATGAAACGGATGTATGATTACATA) were purchased from Sangon Biotech (Shanghai, China). Na_2_SO_4_, tris(hydroxymethyl)aminomethane (Tris), hemin, hydrogen peroxide (H_2_O_2_) of 30% v/v, dimethylsulfoxide (DMSO), tetrabutylammonium hexafluorophosphate (Bu_4_NPF_6_), 2,2′-azino-bis(3-ethybenzthiazoline-6-sulfonic acid) (ABTS), Terephthalic acid (TA), 1,3-disphenylisobenzofuran (DPBF) and acetonitrile were purchased from Adamas Reagent. Ltd. (Shanghai, China). Dopamine hydrochloride (AR, 98%) was purchased from J&K Scientific Ltd. Ce6 was purchased from Macklin Biochemical Co., Ltd (Shanghai, China). Cell counting Kit-8 (CCK-8) was purchased from Dojindo Chemical (Shanghai, China). 2′,7′-Dichlorodihydrofluorescein diacetate (DCFH-DA) and 2′,7′-Dichlorodihydrofluorescein (DCFH) were purchased from Heowns Biochemical Technology Co., Ltd. (Tianjin, China). Macrophage colony-stimulating factor 1 (M-CSF) and Granulocyte-macrophage colony-stimulating factor (GM-CSF) were purchased from Sinobiological. JNK, p38, and NK-κB were purchased from Santa Cruz. phospho-JNK, phospho-NK-κB, and phospho-p38 were purchased from Affinity Bioscience. All fluorophore-conjugated anti-mouse antibodies for flow cytometry assay and the mouse uncoated ELISA kits were obtained from Thermo Fisher Scientific.

**Characterization**

Transmission electron microscopy (TEM) and scanning transmission electron microscopy (STEM) images were obtained using a Talos F200S (ThermoFisher Scientific, Netherlands) instrument with an acceleration voltage of 200 kV to observe the size, morphology, and integrity of the nanosheets. Atomic force microscope (AFM) measurement was performed on an MEP-3D-BIO system (Asylum Research, US). X-ray photoelectron spectroscopy (XPS) was recorded on an ESCALAB250Xi system (Thermo, US). The absorption spectra were measured using a Specord 210 plus UV-Vis Spectrophotometer (Analytikjena, Germany). The fluorescence emission of TAOH was measured by using a fluorescence spectrophotometer (RF-6000, Shimadzu). Absorbance in CCK-8 assay was measured by a microplate reader (Thermo). The electrochemical property of the samples was investigated using a CHI600D electrochemical workstation. Electron spin resonance (ESR) spectra were obtained at room temperature on a Bruker E500 spectrometer. DMPO was used as a spin trap. Fluorescence images were acquired in a confocal laser scanning microscopy (TCS SP8 DIVE, Leica). The concentration of iron element was measured using an inductively coupled plasma-atomic emission spectrometer (ICP-AES, Vista AX, Varian, USA). The fluorescence signal of Cy5.5 and DCFH in vivo was detected by a spectrum in vivo imaging system (IVIS) Lumina III imaging system (PerkinElmer).

Iron contents in G4s/Ce6/Hemin complexes and PDANFs were measured using inductively coupled plasma (ICP, Agilent 5110). The results showed that each milligram of G4s/Ce6/Hemin complexes and PDANFs contained 470 ng and 93 ng of iron, respectively, corresponding to 5.5 μg and 1.1 μg of hemin per milligram of G4s/Ce6/Hemin complexes and PDANFs. Thus, in this study, a mass concentration ratio of 1:5 (G4s/hemin/Ce6 complex to PDANFs) was employed for both *in vitro* and *in vivo* experiments.

**Synthesis of DNA-PDANs**

For DNA-PDANs formation, dopamine and salmon sperm DNA were mixed at a mass ratio of 1: 0.125 and homogenized on a rotary mixer at 30 rpm for 5 min. Tris buffer was then added, and the reaction was continued overnight on the rotary mixer. The resulting nanoparticles were collected by centrifugation at 12,000 rpm for 10 min, washed twice with pure water, and redispersed in ultrapure water.

**Synthesis of PDANSs**

Briefly, the linear template was phosphorylated and mixed with a primer at a 4:1 volume ratio. The mixture was then heated to 95°C for 5 min and gradually cooled to 25°C. Following annealing, the annealed product was mixed with T4 DNA ligase (30 U μL⁻¹), human recombinant albumin, and T4 ligase buffer at a volume ratio of 25:6:10:10, and incubated at 16°C for 4 h. For the amplification step, the resulting circular template was mixed with Phi29 buffer, human recombinant albumin, dNTP mix (2 mM), Phi29 DNA polymerase with the ratio of 10:4:4:8:1. The reaction was carried out at 30°C for 20 min and terminated by heating at 65°C for 10 min to inactivate phi29 DNA polymerase. The product obtained from RCA was purified by ethanol precipitation.

For PDANSs formation, dopamine and purified RCA product were mixed at a mass ratio of 1: 0.125 and homogenized on a rotary mixer at 30 rpm for 5 min. Tris buffer was then added, and the reaction was continued overnight on the rotary mixer. The resulting nanoparticles were collected by centrifugation at 12,000 rpm for 10 min, washed twice with pure water, and redispersed in ultrapure water.

**The catalytic activity assays**

*Electrochemistry measurements:* Cyclic voltammetry (CV) measurements were conducted using a sample film-coated glassy carbon electrode (GCE) as the working electrode, Pt wire as the counter electrode, and Ag/AgCl as the reference electrode in a 0.1 M Bu_4_NPF_6_ acetonitrile solution. A potential was applied from -1.2 V to 0 V with a scan rate 0.1 V s^-1^.

Potential-limited (±2 V vs. Ag/AgCl) galvanostatic discharge measurements of these PDANFs-coated electrodes were carried out at a current of 0.1 μA in 0.5 M Na_2_SO_4_ buffer (pH = 7.0).

*H_2_O_2_ generation testing:* A H_2_O_2_ assay kit was used to evaluate the H_2_O_2_ generation activity of PDANFs, which is based on the oxidation of ferrous ions (Fe^2+^) to ferric ions (Fe^3+^) by peroxides and the Fe^3+^ then combine a dye-xylenol orange to form a purple-colored complex with the maximum absorbance at 560 nm measurable. In brief, PDANFs (10 μg) were incubated with 1 mL of detection solution for sufficient O_2_ or N_2_ supply. After 10 min incubation, the colour change was imaged by digital camera and measured by UV-Vis Spectrophotometer.

*O2˙ˉ generation testing:* DPBF was adopted as a chemical probe for O2**˙**ˉ generation because it could be oxidative degraded in the presence of O2**˙**ˉ with gradual decline of its absorption at 410 nm. PDANFs (10 μg) were dispersed in DPBF solutions (100 μM) and bubbed with O_2_ or N_2_ for 10 min. The absorption spectra of suspensions were monitored using UV-Vis Spectrophotometer.

*Peroxidase mimic activity assay:* G4s nanowires/Ce6/Hemin complexes (20 μg) and PDANFs (100 μg) were respectively dispersed in 1 mL N_2_-saturated buffer (phosphate buffered saline (PBS), pH 6.5), and then, 10 μL ABTS (160 mM) and 10 μL H_2_O_2_ (160 mM) were added into the suspension. The catalytic activity was investigated by the UV-vis absorption spectra (652 nm) within 10 min at room temperature. G4s nanowires/Ce6/Hemin complexes and PDANFs with the same stoichiometry of iron were used in this experiment according to the ICP measurements.

*Cascade reaction activity assay:* TA was used as a fluorescence probe for tracking •OH because it could capture •OH and generate 2-hydroxy terephthalic acid (TAOH), which emitted unique fluorescence around 425 nm. G4s nanowires/Ce6/Hemin complexes and PDANFs were respectively dispersed in TA solution (1 mL, final concentration: 0.5 mM, pH 6.5), respectively. After bubbling N_2_, air, or O_2_ into the suspensions for different times, the fluorescence emission of the resulting solutions was measured by using a fluorescence spectrophotometer.

**Cell culture**

The murine breast cancer cells (4T1 cells) and murine macrophage cells (RAW 264.7 cells) were cultured in a growth medium (DMEM high-glucose) supplemented with FBS, streptomycin (100 units mL^-1^), and penicillin (100 units mL^-1^). The cells were cultured at 37℃ in a humidified atmosphere with 5% CO_2_ in air.

Mouse BMMs and BMDCs were isolated from C57BL/6 mice as previously reported^1^. The bone marrow was flushed from femur and tibia bones and then cultured in α-modified minimal complete medium complete medium containing 10% FBS and penicillin plus streptomycin. To obtain pure BMMs, adnexal-free cells were collected and cultured in complete α-MEM plus M-CSF (25 ng mL^-1^). After 5 days of culture, the attached cells were used for subsequent experiments. BMMs were divided into three different groups and stimulated for 24 h in the presence of either murine IL-4 (20 ng mL^-1^) to polarize them to an M2 phenotype or LPS (100 ng mL^-1^) to polarize to them to an M1 phenotype. Untreated BMMs constituted the naïve group (M0s). In the majority of the experiments described in this study, the unstimulated BMMs (M0s) would be compared against pre-polarized M1s (serving as negative control) and per-polarized M2s (serving as positive control). To obtain pure BMDCs, the bone marrow was cultured in α-MEM supplemented with GM-CSF (25 ng mL^-1^) and IL-4 (10 ng mL^-1^). The medium was half-replaced every 2 days. On the fifth day of culture, the medium was gently pipetted; immature BMDCs cells that were suspended in the medium and loosely adhered to the flask were all collected for further use.

**Supplementary figures**

**Figure S1**. Scheme of the HCR-mediated G4 nanowires.

**Figure S2.** TEM images of PDANFs synthesized at varying dopamine/G4 nanowires mass ratio: 1 : 0.03125 (A), 1 : 0.125 (B), 1 : 1 (C).

**Figure S3.** CV of different sample film-coated glassy carbon electron (GCE) in 0.1 M Bu_4_NPF_6_ acetonitrile solution at 0.1 V s^-1^.

**Figure S4.** Proposed mechanism of the self-assembled G4s nanowires actin as deposition sites for PDA polymerization.

**Figure S5**. (A) Optimized model for theoretical docking study of PDA oligomer and G4s. (B) The snapshots of the complex at 10, 50, and 100 ns during the molecular dynamics simulations. (C) RMSD change over molecular dynamics simulation. (D) SASA change over molecular dynamics simulation. (E) The binding free energy decomposition analyzed by using MM-PBSA method.

**Figure S6**. Mechanism of O_2_˙ˉ dismutation.

**Figure S7**. (A) The synthesis mechanisms of PDANSs. (B) TEM images showing the morphology and composition of PDANSs, including the bright-field TEM images, a dark-field TEM image, and the corresponding element mapping data. (C) Comparison of HRP-like catalytic activity between PDANFs and PDANSs.

**Figure S8**. (A) O_2_ concentration-dependent absorbance variations of ABDA solutions (10 μM) at 380 nm in the presence of PDANFs-ox. (B) O_2_ concentration-dependent absorbance variations of the H_2_O_2_ detection solution in the presence of PDANFs. (C) O_2_ concentration-dependent fluorescence intensity variations of TA solutions (0.5 mM) in the presence of PDANFs.

**Figure S9**. Long-term stability study of semiquinone radicals in PDANFs by monitoring the •OH generation efficiency (mean ± SD; n = 3).

**Figure S10**. High-resolution XPS spectra of O1s of oxidized PDANFs.

**Figure S11**. Effects of different formulation ratios (dopamine/G4 nanowires mass ratio of 1:0.03125 (A), 1:0.125 (B), and 1:1 (C)) on the ROS-generating capacity of the nanofibers.

**Figure S12**. (A) The DLS characterizations of PDANFs in FBS for 1 day and 30 days. (B) TEM image of PDANFs in FBS for 30 days.

**Figure S13**. pH-dependent fluorescence intensity increases of TA at 425 nm in the presence of GH complexes.

**Figure S14**. Relative fluorescence intensity of DCFH upon addition of PDANFs or PDANFs-ALG. The dispersion was treated with NIR irradiation and subsequently incubated under hypoxic conditions (1% O_2_) for an additional 0.5 hours, and then the fluorescence intensity was measured using a fluorescence spectrophotometer. Error bars are based on the standard error of the mean (n = 3).

**Figure S15**. Quantitative analysis of time-dependent fluorescence reduction based on flow cytometry results.

**Figure S16**. CLSM images of ROS signal in cells after different treatments.

**Figure S17**. Viability of L929 cells incubated with PDANFs at various concentration (from 1 μg mL^-1^ to 20 μg mL^-1^) for 24 h (*n* = 5).

**Figure S18**. Cell viabilities of 4T1 cells after various treatment. The experiments were conducted in the presence of 20 μg mL^-1^ PDANs, 4 μg mL^-1^ G4s/Ce6 complexes, or 20 μg mL^-1^ PDANFs (n=5).

**Figure S19**. Fluorescence live (green)/dead (red) cell images of 4T1 cells after various treatment.

**Figure S20.** (A) Representative optical images of 4T1 cells with different treatments. The red arrows show the bubbles from the plasma membrane. (B) Western blot analysis of pyroptosis-related markers of 4T1 cells after different treatments. (C, D) Quantification of GSDMD-N and cleaved caspase-3 proteins, respectively.

**Figure S21.** Density plots and the corresponding data for cell apoptosis analysis after 4T1 with different treatments.

**Figure S22**. Extracellular release amount of HMGB1 from 4T1 cells after various treatments.

**Figure S23**. Schematic diagram of *in vitro* co-incubation system of 4T1 cells and BMDCs or BMMs. Before co-incubation, 4T1 cells were treated with nanomaterial-ALG hydrogel plus NIR irradiation.

**Figure S24**. Density plots and the corresponding data for polarization analysis after BMMs treatment with PDANFs-ox-ALG hydrogel and varying concentrations of H_2_O_2_.

**Figure S25**. Density plots and the corresponding data for cell apoptosis analysis after BMMs treatment with PDANFs-ox-ALG hydrogel and varying concentrations of H_2_O_2_.

**Figure S26**. Relative protein expression statistic of p-JNK (A), p-NF-κB (B), and p-p38 (C).

**Figure S27**. *In vivo* fluorescence images of tumor-bearing mice at 72 h after intratumoral injection of Cy5.5-PDANFs/ALG hydrogel.

**Figure S28**. Confocal fluorescence micrographs of tumour slices collected from mice injected with Cy5.5-PDANFs/ALG hydrogel. Cell nuclei were stained with DAPI (blue).

**Figure S29**. (A) Immunofluorescence images of tumor slices. Scale bar: 50 μm. Red represented HIF-1α, and blue represented DAPI. (B, C) *In vivo* fluorescence images and quantitative results of ROS level at tumors with different sizes.

**Figure S30**. (A) Time-resolved IR thermal images of 4T1 tumor-bearing mice under the applied NIR at post-injection of PBS-hydrogel and PDANs-hydrogel. (B) Mean tumor site temperature as a function of irradiation time.

**Figure S31.** Histological images of H&E-stained major organ slices harvested from the mice after different treatments.

**Figure S32.** Hematology analysis of PBS and PDANFs treated mice. The tested indexed include white blood cells (WBC), neutrophils (NEUT), lymphocyte (LYM), mixed cell count (MXD), hemoglobin (HGB), red blood cells (RBC), hematocrit (HCT), mean corpuscular volume (MCV), mean corpuscular hemoglobin (MCH), mean corpuscular hemoglobin concentration (MCHC), platelet (PLT), plateletcrit (PCT), mean platelet volume (MPV). The yellow filled areas indicate the normal range of hematology indicators. Error bars are based on the standard error of the mean (mean) (*n = 5*).

**Figure S33.** Representative immunofluorescence images of tumors showing macrophage polarization in tumor tissue following various treatments.

**Figure S34**. (A) Bilateral tumor xenograft modeling and treatment. Treated mice received hydrogel implants only on the primary tumor. (B and C) Primary and distant tumor growth curves in different treated mice. Error bars are based on the standard error of the mean (*n* = 5). ***p* < 0.01, ****p* < 0.001, and *N.S.* represents no significant different determined by Student’s *t*-test.

**Figure S35**. Representative immunofluorescent staining images of distant tumors showing DAPI (blue), CD4^+^ (green), CD8^+^ (red) cells infiltration for different treatment groups.

**Figure S36**. The photographs and H&E staining of the metastatic foci of 4T1 lung metastatic tumors.

**Figure S37**. (A) The proportions of various immune cell types and 4T1 cell in tumors. (B) The proportions of various macrophages in tumors. (C) The proportions of various CD8^+^ T cells in tumors.

**References**

[1]	Liu, X.; Li, F.; Dong, Z., et al., Metal-polyDNA nanoparticles reconstruct osteoporotic microenvironment for enhanced osteoporosis treatment. *Sci. Adv. 9* (31), eadf3329.
